# Methylglyoxal couples metabolic and translational control of Notch signalling in mammalian neural stem cells

**DOI:** 10.1038/s41467-020-15941-2

**Published:** 2020-04-24

**Authors:** Deivid Carvalho Rodrigues, Emily M. Harvey, Rejitha Suraj, Sarah L. Erickson, Lamees Mohammad, Mengli Ren, Hongrui Liu, Guiqiong He, David R. Kaplan, James Ellis, Guang Yang

**Affiliations:** 1 Program in Developmental & Stem Cell Biology, Hospital for Sick Children, Toronto, ON M5G 0A4 Canada; 2 Department of Medical Genetics, University of Calgary, Calgary, AB T2N 4N1 Canada; 3 Department of Biochemistry and Molecular Biology, University of Calgary, Calgary, AB T2N 4N1 Canada; 4 Alberta Children’s Hospital Research Institute, Calgary, AB T2N 4N1 Canada; 5 Institute of Neuroscience, Chongqing Medical University, Chongqing, 400016 China; 6 Program in Neurosciences & Mental Health, Hospital for Sick Children, Toronto, ON M5G 0A4 Canada; 7 Department of Molecular Genetics, University of Toronto, Toronto, ON Canada

**Keywords:** Developmental neurogenesis, RNA metabolism, Translation, Neural progenitors, Neural stem cells

## Abstract

Gene regulation and metabolism are two fundamental processes that coordinate the self-renewal and differentiation of neural precursor cells (NPCs) in the developing mammalian brain. However, little is known about how metabolic signals instruct gene expression to control NPC homeostasis. Here, we show that methylglyoxal, a glycolytic intermediate metabolite, modulates Notch signalling to regulate NPC fate decision. We find that increased methylglyoxal suppresses the translation of *Notch1* receptor mRNA in mouse and human NPCs, which is mediated by binding of the glycolytic enzyme GAPDH to an AU-rich region within *Notch1* 3ʹUTR. Interestingly, methylglyoxal inhibits the enzymatic activity of GAPDH and engages it as an RNA-binding protein to suppress *Notch1* translation. Reducing GAPDH levels or restoring Notch signalling rescues methylglyoxal-induced NPC depletion and premature differentiation in the developing mouse cortex. Taken together, our data indicates that methylglyoxal couples the metabolic and translational control of Notch signalling to control NPC homeostasis.

## Abstract (web summary)

Gene regulation and metabolism co-ordinate self-renewal and differentiation of neural precursors (NPCs) in the developing brain. Here the authors show that methylglyoxal, a glycolytic intermediate metabolite, promotes GADPH-dependent translational repression of Notch1, thereby promoting NPC differentiation.

## Introduction

During the development of the mammalian brain, neural precursor cells (NPCs) self-renew and differentiate to give rise to appropriate numbers of neurons^1^. Key to this balance of self-renewal and differentiation is the crosstalk between gene expression and metabolism, two fundamental processes that co-ordinate NPC fate decision^2–4^. The expression of metabolic genes is known to be tightly controlled to support the metabolic shift during NPC differentiation. For example, activation of Notch signalling in NPCs induces the expression of pro-proliferative genes (e.g. basic helix-loop-helix transcription factor *Hes1*) to maintain the self-renewal of NPCs while at the same time upregulating the expression of glycolytic enzyme genes (e.g. hexokinase 2 and lactate dehydrogenase) that are required by NPCs for energy production and biosynthesis^5,6^. On the other hand, during neurogenesis NPCs express proneural genes to induce differentiation and downregulate the expression of glycolytic enzymes to support the metabolic transition from glycolysis to mitochondrial oxidative phosphorylation as the primary energy source of neurons^5,6^. Accumulating evidence suggests that NPC metabolism is not a simple adaptation to different cellular states but instead plays a more direct role in regulating self-renewal and differentiation. Although NPCs rely on glycolysis, their mitochondria exhibit an elongated morphology and are functional, with a forced metabolic switch to mitochondrial oxidative phosphorylation enhancing their differentiation^7,8^. These observations reveal the reciprocal nature of the relationship between metabolism and gene expression critical for NPC fate decision. However, while the pathways that regulate metabolic gene expression are well described, the signals and mechanisms that mediate the metabolic feedback control of gene expression for proper NPC fate decision remain poorly understood.

Glycolysis produces a wealth of metabolites. How do these metabolic cues instruct gene expression in NPCs? One mechanism used in the adult murine NPCs is mediated by a cyclic AMP-responsive element-binding protein (CREB)-dependent pathway that senses the level of glucose to direct *Hes1* transcription and thereby the self-renewal of NPCs^9^. A second mechanism may involve glycolytic enzymes acting as RNA-binding proteins (RBPs) to regulate target mRNAs post-transcriptionally^10–12^. For example, glyceraldehyde-3-phosphate dehydrogenase (GAPDH) is a key glycolytic enzyme that catalyzes the conversion of glyceraldehyde-3-phosphate (G3P) into 1, 3-bisphosphoglycerate (1, 3-BPG)^13^. Interestingly, GAPDH can bind to the AU-rich element in the 3ʹ untranslated region (3ʹUTR) of mRNAs and subsequently alter their stability and translation^14^. This dual function of GAPDH is best described in immune cells. In T cells where oxidative phosphorylation serves as the primary energy source, GAPDH functions as an RBP to repress the translation of the interferon γ mRNA^10^. When T cells are activated and switch from oxidative phosphorylation to glycolysis, GAPDH is re-engaged in the glycolytic pathway and no longer represses the translation of interferon-γ mRNA^10^.

What controls the functional switch of metabolic enzymes is still largely unknown. One means of switching may involve feedback or feedforward control of their enzymatic activities by post-translational modifications with intermediate metabolites^15,16^. For example, methylglyoxal, an intermediate metabolite produced from G3P during glycolysis modifies GAPDH in a non-enzymatic manner, leading to inhibition of its enzymatic activities^17^. The competitive binding between the enzyme cofactor nicotinamide adenine dinucleotide (NAD) and RNA to the same domain on GAPDH suggests that its compromised activity for glycolysis may otherwise promote its engagement as an RBP to regulate target mRNAs^18,19^.

We have recently found that an increase in methylglyoxal levels depletes NPC numbers in the developing mouse cortex^20^, raising the possibility that methylglyoxal may serve as a metabolic signal to regulate specific genes for NPC homeostasis by modulating RNA-binding enzymes such as GAPDH. Here, we show that methylglyoxal induces feedback regulation of Notch signalling in NPCs by engaging GAPDH as an RBP. An increase in methylglyoxal levels reduces the enzymatic activity of GAPDH and promotes its binding to *Notch1* mRNA in NPCs. This leads to the translational repression of *Notch1* mRNA and a reduction in Notch signalling, ultimately causing premature neurogenesis. This study provides a mechanistic link for the metabolic regulation of gene expression in NPC homeostasis.

## Results

### Excessive methylglyoxal depletes neural precursors

**Fig. 1:**
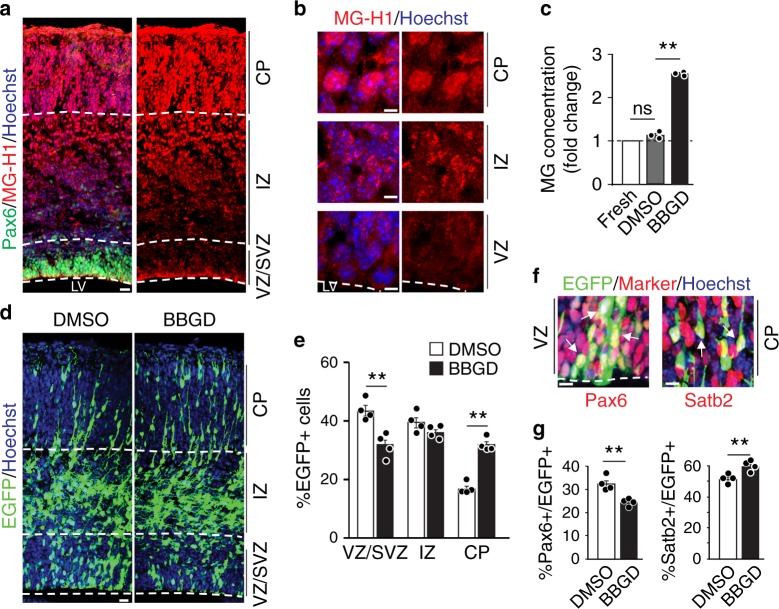
Inhibition of Glo1 perturbs the maintenance of NPCs in the developing cortex. **a**, **b** E16.5 coronal cortical sections immunostained for MG-H1 (red). In **a**, sections were costained for Pax6 (green), and the ventricular surface and cortical boundaries between VZ/SVZ, IZ and CP are labelled with dotted white lines. **b** High-magnification images of cells in the VZ, IZ and CP from sections as in **a**. *n* = 2 experiments. **c** Relative methylglyoxal levels measured in cortical tissues isolated from the E13.5 cortex (fresh) or treated with BBGD or DMSO vehicle alone. *n* = 3 experiments. **d**–**g** BBGD or DMSO was injected into lateral ventricles of E13.5 cortices, followed by the electroporation with EGFP. Coronal cortical sections were analyzed 3 days later. Sections were immunostained for EGFP (green, **d**, **f**), and the relative location of EGFP+ cells was quantified (**e**). *n* = 4 embryos each. **f** Images of the VZ or CP of electroporated sections that were costained for Pax6 (red, left) or Satb2 (red, right). Arrows denote double-labelled cells. **g** Quantification of sections as in **f** for the proportion of EGFP+ cells that were also positive for Pax6 or Satb2. *n* = 4 embryos each. Sections were counterstained with Hoechst 33258 (blue, **a**, **b**, **d** and **f**). LV lateral ventricle, VZ ventricular zone, SVZ subventricular zone, IZ intermediate zone, CP cortical plate. Scale bars, 50 μm in **a** and **d**, 10 mm in **f** and 5 mm in **b**. Data are presented as mean values ± SEM and analyzed using two-tailed, unpaired students *t*-test with Bonferroni correction. **p < 0.01, ns = *p* > 0.05. Source data and p-values are provided as a “Source Data file”.

We have previously shown that methylglyoxal-metabolizing enzyme glyoxalase 1 (Glo1) maintains NPC homeostasis, thereby preventing premature neurogenesis in the developing murine cortex^20^. To determine whether Glo1 controls NPC differentiation by enzymatically modulating methylglyoxal, we initially assessed methylglyoxal-adduct levels in NPCs and neurons in the cortex^21,22^. Immunostaining of embryonic day 16.5 (E16.5) cortical sections for a major methylglyoxal-adduct MG-H1 showed only weak immunoreactivity in the cytoplasm of Pax6+ radial precursors in the ventricular and subventricular zones (VZ/SVZ) (Fig. 1a, b, Supplementary Fig. 1a). MG-H1 production was gradually increased in newborn neurons migrating in the intermediate zone (IZ) and became highly enriched in the cortical plate (CP), where it accumulated in the nuclei of neurons expressing neuronal markers βIII-tubulin (cytoplasmic) and Brn1 (nuclear) (Fig. 1a, b, Supplementary Fig. 1a). The gradual increase in methylglyoxal levels from NPCs to neurons was consistent with a previous study^23^ and is in agreement with the higher expression level of Glo1 in NPCs than in neurons^20^. We next manipulated Glo1 enzymatic activity using S-p-bromobenzylglutathione diethyl ester (BBGD), a cell-permeable and reversible Glo1 inhibitor^24^. As expected, upon incubation with BBGD, methylglyoxal levels were significantly elevated in isolated E13.5 cortical tissues (Fig. 1c). We then injected BBGD into the lateral ventricle at E13.5 followed by in utero electroporation of a plasmid encoding nuclear EGFP to label and track NPCs and the neurons they give rise to. The reversible effect of BBGD allows the manipulation of NPCs adjacent to the lateral ventricle, with a minimal impact on migrating newborn neurons in the IZ. Cortical sections were immunostained for EGFP and cell-type-specific markers three days after treatment. We found that BBGD exposure led to a reduction of EGFP+ cells in the VZ/SVZ (Fig. 1d, e). In contrast, the proportion of EGFP+ cells in the CP was increased, with no change in proportions in the IZ (Fig. 1d, e). In line with the altered cell distribution, we found fewer EGFP+ cells that also expressed the radial precursor marker Pax6, and more EGFP+ cells expressing the neuronal marker Satb2 in cortices exposed to BBGD (Fig. 1f, g). To further confirm that the alterations of NPCs were due to an aberrant increase in methylglyoxal, we labeled proliferating NPCs with bromodeoxyuridine (BrdU) followed by injection of PBS or methylglyoxal into the lateral ventricle. Two days later, we found fewer BrdU+ cells in the VZ/SVZ as well as fewer BrdU+, Pax6+ radial precursors in the cortex received methylglyoxal (Supplementary Fig. 1b–g). These results indicate that an increase in methylglyoxal shifts the balance of NPC homeostasis towards neurogenic differentiation.

### Methylglyoxal regulates Notch signalling to affect NPCs

**Fig. 2:**
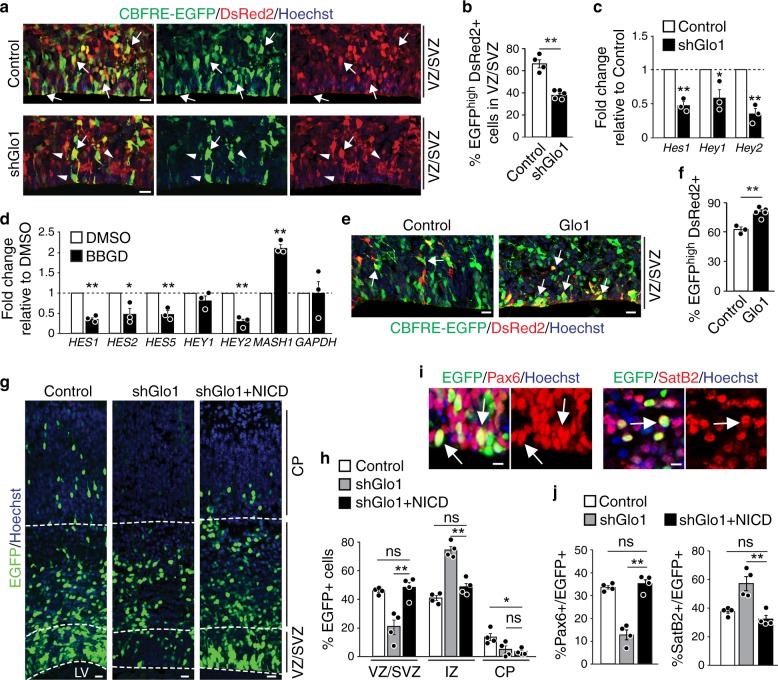
Methylglyoxal perturbs NPC by suppressing Notch signalling. **a**, **b** E13.5 cortices were co-electroporated with CBFRE-EGFP and DsRed2, and control or Glo1 (shGlo1) shRNAs and analyzed two days later. **a** Images of sections immunostained for EGFP (green) and DsRed2 (red). Arrows denote double-labelled cells, and arrowheads denote cells with reduced Notch signalling. **b** Quantification of sections as in **a** for EGFP+, DsRed2+ cells. *n* = 4 and 5 embryos each. **c** qRT-PCR analysis of FACS sorted EGFP+ cells from cortices co-electroporated with EGFP and control or *Glo1* shRNAs. *n* = 3 experiments. **d** qRT-PCR analysis of low passage hNPCs treated with BBGD or DMSO for 48 h. *n* = 3 experiments. **e**, **f** E13.5 cortices were co-electroporated with CBFRE-EGFP and DsRed2 plus empty vector control or a plasmid expressing Glo1 (Glo1) and analyzed two days later. Images (**e**) and quantification (**f**) of EGFP (green) and DsRed2 (red) double-positive cells. *n* = 3 and 5 embryos each. **g**–**j** A low dose of plasmids (0.5 µg µl^−1^) expressing NICD was co-electroporated with *Glo1* shRNA and EGFP into E13.5 cortices. Cortical sections were analyzed three days later. **g** Images of sections immunostained for EGFP (green). Dotted white lines denote the ventricular surface and boundaries between VZ/SVZ, IZ, and CP. **h** Quantification of the relative localization of EGFP + cells. *n* = 4 embryos each. **i** Images of the VZ or CP of electroporated sections that were immunostained for EGFP (green) and Pax6 (red, left panels) or Satb2 (red, right panels). Arrows denote double-labelled cells. **j** Quantification of the proportion of EGFP+ cells that were also positive for Pax6 or Satb2. *n* = 4 embryos each. LV lateral ventricle, VZ ventricular zone, SVZ subventricular zone, IZ intermediate zone, CP cortical plate. Scale bars, 25 µm in **a**, **e** and **g**; 10 µm in **i**. Data are presented as mean values ± SEM and analyzed using two-tailed, unpaired students *t*-test with Bonferroni correction. **p < 0.01, *p < 0.05, ns = *p* > 0.05. Source data and *p*-values are provided as a “Source Data file”.

Notch signalling plays a crucial role in stem-cell maintenance^5^. Perturbing components of Notch signalling leads to NPC depletion and aberrant neurogenesis characterized by NPC apical detachment and mislocalization in the developing cortex^25^, which is phenocopied by *Glo1* knockdown^20^. Therefore, we asked whether methylglyoxal alters Notch signalling in NPCs, using a Notch signalling reporter that contains the responsive element of the canonical Notch effector c-promoter binding factor 1 (CBF1) upstream of the EGFP gene (CBFRE-EGFP)^25,26^. We co-electroporated the Notch signalling reporter with control or *Glo1* shRNA plasmids into E13.5 cortices and a plasmid encoding DsRed2 driven by the constitutive CMV promoter, used as an internal transfection control. After two days, almost 70% of DsRed2+ cells in the VZ/SVZ of the control cortex expressed EGFP, indicating active Notch signalling in the transfected cells (Fig. 2a, b). However, following *Glo1* knockdown, this number was reduced to less than 40%, suggesting that Notch signalling is suppressed by excessive methylglyoxal induced by *Glo1* knockdown. To further assess the changes in Notch signalling, we examined the mRNA levels of downstream Notch targets in control and *Glo1* knockdown cells. To this end, we co-electroporated control or *Glo1* shRNAs with an EGFP plasmid into E13.5 cortices and collected EGFP+ cells 2 days later by fluorescence-activated cell sorting (FACS) for quantitative real-time polymerase chain reaction (qRT-PCR) analysis. The mRNA levels of the Notch1 targets, *Hes1*, *Hey1,* and *Hey2*, were significantly reduced following *Glo1* knockdown (Fig. 2c), suggesting a reduction of Notch signalling. To confirm these findings, we also examined the effect of Glo1 inhibition on Notch signalling in a relatively homogenous NPC population, in vitro cultured human embryonic stem-cell (H9)-derived NPCs (hNPCs). Treatment with BBGD caused a significant increase in methylglyoxal levels (Supplementary Fig. 1h) and reduced the mRNA levels of *HES1*, *HES2, HES5,* and *HEY2* genes (Fig. 2d). The expression of *MASH1*, which is repressed by the HES protein family, was correspondingly upregulated (Fig. 2d). Moreover, knockdown of *GLO1* in hNPCs caused an increase in intracellular methylglyoxal levels (Supplementary Fig. 2a–d), accompanied by a reduction in expression of Notch1-responsive genes (Supplementary Fig. 2e). These results indicate that excessive methylglyoxal reduces Notch signalling in NPCs. Further support for this conclusion came from the direct application of exogenous methylglyoxal to hNPCs, which also increased intracellular methylglyoxal levels (normalized fold change, 10.16 ± 1.80; *p* < 0.01; *n* = 3) and suppressed Notch1-responsive genes (Supplementary Fig. 2f).

To determine if physiological levels of methylglyoxal regulate Notch signalling, we overexpressed *Glo1* in the cortex by in utero electroporation to deplete intracellular methylglyoxal and at the same time, co-electroporated the Notch signalling reporter. The analysis of the cortex after two days showed that *Glo1* overexpression significantly increased the number of VZ/SVZ cells that had active Notch signalling (Fig. 2e, f), in agreement with a concomitant increase in NPC numbers (Supplementary Fig. 2g–j). Consistently with these results, ectopic expression of human *GLO1* in cultured hNPCs reduced methylglyoxal levels (Supplementary Fig. 1h) and caused an increase in the expression of Notch1-responsive genes (Supplementary Fig. 2k).

Taken together, our results indicate that methylglyoxal regulates Notch signalling in NPCs and raise the possibility that impaired Notch signalling might account for the depletion of cortical NPCs induced by increased methylglyoxal. If so, then restoring Notch signalling in NPCs might antagonize the effect of methylglyoxal and rescue *Glo1* knockdown-induced NPC depletion. Notch signalling is initiated when the Notch1 receptor binds to its ligands, followed by protease cleavage and release of the Notch intracellular domain (NICD) that translocates into the nucleus to activate downstream targets^5,26^. To test our hypothesis, we constitutively activated Notch signalling by ectopically expressing NICD in the E13.5 cortex in which *Glo1* was also knocked down. After 3 days, our analysis of cortices showed that NICD expression completely reversed the phenotype induced by *Glo1* knockdown, resulting in significantly more EGFP+ cells in the VZ/SVZ as well as EGFP + Pax6+ radial precursors and a reduction in Satb2+ neurons (Supplementary Fig. 3a–c). Given the important role of Notch signalling in NPC maintenance, we wondered whether the phenotypic rescue by NICD was due to an unspecific overactivation of Notch signalling. We therefore titrated the dose of NICD used in electroporation to a level at which NICD itself did not change cortical development (Supplementary Fig. 3d–f). This low dose of NICD was sufficient to normalize the aberrant distribution of EGFP+ cells in the VZ/SVZ induced by *Glo1* knockdown (Fig. 2g, h) and rescued the proportions of EGFP+, Pax6+ radial precursors and Satb2+ neurons to control levels (Fig. 2i, j, Supplementary Fig. 3g). These results demonstrate that the downregulation of Notch signalling mediates methylglyoxal-induced NPC depletion and premature neurogenesis. Interestingly, NICD expression did not rescue the reduction of EGFP+ cells in the CP (Fig. 2g, h, Supplementary Fig. 3a, b), suggesting that methylglyoxal regulates neuronal migration independent of Notch signalling.

### Methylglyoxal regulates translation of *Notch1* mRNA

**Fig. 3:**
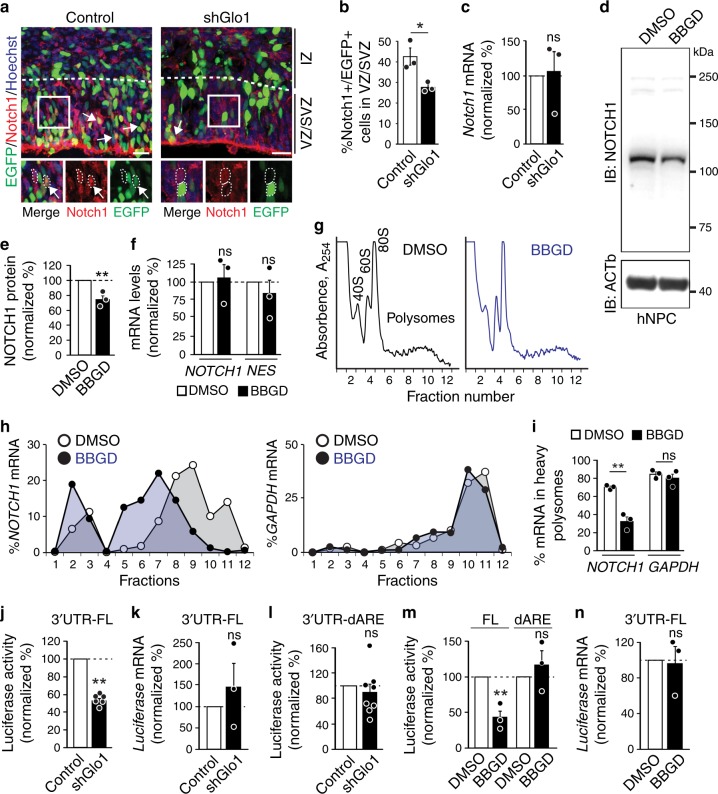
Methylglyoxal suppresses *Notch1* mRNA translation. **a**–**c** E13.5 cortices were co-electroporated with nuclear EGFP and control or Glo1 (shGlo1) shRNAs and analyzed two days later. Images (**a**) and quantification (**b**) of EGFP + cells (green) also positive for Notch1 (red). White boxes in **a** show higher magnification at the bottom. Arrows denote double-labelled cells. *n* = 3 embryos each. **c** qRT-PCR analysis for *Notch1* mRNA in FACS sorted EGFP+ cells from cortices as in **a**. *n* = 3 embryos each. **d**–**i** hNPCs were treated with DMSO or BBGD for 48 h. Images (**d**) and quantification (**e**) from western blots of hNPCs probed for NOTCH1 and ACTb. *n* = 3 experiments. **f** qRT-PCR analysis for *NOTCH1* and *NESTIN* (*NES*) mRNAs in hNPCs. *n* = 3 experiments. **g** Polysome profile of hNPCs. Fractions corresponding to 40 S and 60 S ribosome subunits, the 80 S monosomes and polysomes are labelled. **h** Relative distribution of the *NOTCH1* mRNA (left) and *GAPDH* mRNA (right) across all fractions measured by qRT-PCR. Each point corresponds to the value of each fraction normalized to the total *NOTCH1* or *GAPDH* mRNA. **i** Quantification of the relative enrichment of *NOTCH1* and *GAPDH* mRNAs in the heavy polysome fractions (#8–12). *n* = 3 experiments. **j**–**l** Dual-luciferase reporters containing the full-length (FL) or truncated *Notch1* 3ʹUTR (dARE) were co-electroporated into E13.5 cortices with control or *Glo1* shRNAs. **j** Luciferase activity of the reporter. *n* = 6 embryos each. **k** Luciferase mRNA levels quantified by qRT-PCR. *n* = 3 embryos each. **l** Luciferase activity from the 3ʹUTR-dARE reporter. *n* = 8 embryos each. **m**, **n** hNPCs were transfected with dual-luciferase reporters and treated with DMSO or BBGD for 48 h. **m** Luciferase activity of the reporters.; *n* = 3 experiments. **n** Luciferase mRNA levels quantified by qRT-PCR. *n* = 3 experiments. Scale bars, 25 µm. Data are presented as mean values ± SEM and analyzed using two-tailed, unpaired students *t*-test. **p < 0.01, *p < 0.05, ns = *p* > 0.05. Source data and p-values are provided as a “Source Data file”.

Our data suggest that methylglyoxal may target-specific component(s) of the Notch pathway to regulate NPC homeostasis. Therefore, we asked whether one target could be the Notch1 receptor itself since it initiates the signalling cascade and is under extensive regulation for precise cell fate decision^27–32^. To test whether methylglyoxal regulates *Notch1* expression in NPCs, we electroporated control or *Glo1* shRNAs and a nuclear EGFP plasmid into the E13.5 cortex and examined Notch1 protein expression by immunostaining EGFP+ cells 2 days later. The quantification of Notch1+ cells in VZ/SVZ showed a 35% reduction following *Glo1* knockdown (Fig. 3a, b), in line with our observation of attenuated Notch signalling (Fig. 2a–c). To determine if the reduction in Notch1 protein abundance was due to a decrease in its mRNA levels, we sorted EGFP+ cells from electroporated cortices by FACS and examined *Notch1* mRNA by qRT-PCR. *Glo1* knockdown did not affect *Notch1* mRNA levels (Fig. 3c). Similarly, we found that *GLO1* knockdown caused a reduction in NOTCH1 protein abundance in hNPCs without changing its mRNA levels (Supplementary Fig. 4a–c).

We next examined NOTCH1 protein and mRNA levels in cultured hNPCs treated with BBGD or directly with methylglyoxal. Western blot and qRT-PCR analyses showed that while BBGD and methylglyoxal treatment caused a reduction in NOTCH1 protein abundance (Fig. 3d, e, Supplementary Fig. 4d, e), *NOTCH1* mRNA levels again were unaltered (*NESTIN* mRNA used as a control) (Fig. 3f, Supplementary Fig. 4f). Given that glycolysis is the primary source of intracellular methylglyoxal, we asked whether an increased glycolytic flux induced by high glucose condition could lead to similar changes in hNPCs. However, high glucose medium did not affect methylglyoxal levels in hNPCs (Supplementary Fig. 4g). In contrast, ectopic expression of *GLO1* that reduced methylglyoxal in hNPCs (Supplementary Fig. 1h) caused an increase in NOTCH1 protein abundance (Supplementary Fig. 4h, i) but did not affect *NOTCH1* mRNA levels (Supplementary Fig. 4j).

These results suggest that the regulation of *NOTCH1* expression may occur at the translational level. To test this, we performed polysome profiling to measure mRNA translational status by separating non-translated mRNAs from those being associated with multiple ribosomes (heavy polysomes) for active translation (Fig. 3g). We observed that BBGD treatment did not alter the polysome profile in hNPCs (Fig. 3g), suggesting that global translation was not perturbed by excessive methylglyoxal. This was consistent with the lack of changes seen in total protein synthesis measured by puromycin metabolic incorporation (Supplementary Fig. 4h, i). We then examined the polysomal distribution of *NOTCH1* mRNA by qRT-PCR. BBGD treatment induced a robust shift in *NOTCH1* mRNA towards lighter polysome fractions, with a concomitant increase in fractions containing non-translated mRNA (Fig. 3h, i), indicating reduced translation. On the contrary, the highly expressed *GAPDH* mRNA was similarly engaged in translation in both conditions (Fig. 3h, i). Together, these results indicate that methylglyoxal negatively regulates *NOTCH1* expression in NPCs at the level of translation.

### Suppression of *Notch1* translation requires an AU-rich motif

Translational regulation of *Notch1* mRNA has been described in *C. elegans* during germline development^33,34^, and recently in mouse T cells during thymocyte development^35^. In these cases, the translational regulation of Notch receptors is mediated by the 3ʹUTR of *Notch1* mRNA. Therefore, we asked whether the *Notch1* 3ʹUTR mediated methylglyoxal-induced translational repression. Using luciferase reporter assay, we included the full-length *Notch1* 3ʹUTR downstream of the firefly luciferase gene in a dual-luciferase vector and electroporated it with control or *Glo1* shRNAs into the E13.5 cortex. Analysis of the cortex 2 days later showed that *Glo1* knockdown markedly suppressed firefly luciferase activity (Fig. 3j), while luciferase mRNA levels remained unchanged (Fig. 3k), suggesting the presence of a translational regulatory component within the *Notch1* 3ʹUTR. The AU-rich element within the *Notch1* 3’ UTR mediates the translational regulation of *Notch1* by interacting with RBPs in T cells^35^. We found that the deletion of this AU-rich region in the *Notch1* 3ʹUTR completely abolished *Glo1* knockdown-induced reduction in luciferase activity in the cortex (Fig. 3l). Similarly, in cultured hNPCs, BBGD treatment significantly suppressed the luciferase activity from the reporter with the full-length *Notch1* 3ʹUTR but not the construct lacking the AU-rich region (Fig. 3m, n), suggesting that the AU-rich region present in the *Notch1* 3ʹUTR mediates the translational regulation of *Notch1* mRNA.

### GAPDH binds to the *Notch1* 3ʹUTR to represses its translation

Given the involvement of the AU-rich region, RBP(s) may interact with this region to mediate methylglyoxal-induced translational repression of *Notch1* mRNA. We reasoned that methylglyoxal might modulate this interaction by changing the availability or enzymatic activity of an RBP since the post-translational addition of methylglyoxal moieties can change protein stability and functions^21,36–38^. We focused our search of RBPs to those known to bind AU-rich elements and to be modified by methylglyoxal. The glycolytic enzyme GAPDH is a known target of methylglyoxal modification, and this modification inhibits its enzymatic activity for glycolysis^17^. Interestingly, when its enzymatic function in glycolysis is disengaged in T cells, GAPDH acts as an RBP to suppress the translation of interferon γ mRNA after binding an AU-rich sequence within interferon γ 3ʹUTR^10^. This raises the possibility that GAPDH mediates the effect of methylglyoxal on *Notch1* translation in NPCs.

**Fig. 4:**
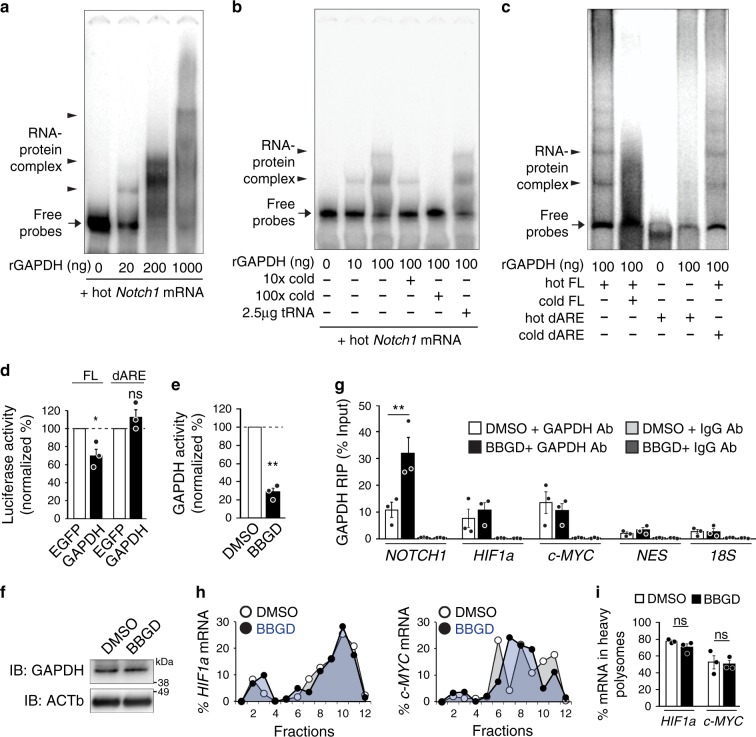
GAPDH binds *Notch1* mRNA 3ʹUTR in response to excessive methylglyoxal and regulates *Notch1* translation. **a**–**c** REMSA was performed using the labelled (hot) full-length (FL) or AU-rich element-deleted (dARE) *Notch1* 3ʹUTR RNA probe in the presence of incremental amounts of recombinant GAPDH protein and the presence of unlabeled (cold) specific or unspecific RNA probes. Arrows and arrowheads indicate free RNA probes and RNA-GAPDH complexes, respectively. *n* = 3 experiments. **d** Luciferase activity from a reporter containing the full-length (FL) or AU-rich element-deleted (dARE) *Notch1* 3ʹUTR co-transfected with a plasmid expressing EGFP control or GAPDH in HEK293 cells. Firefly luciferase activity values were normalized to Renilla luciferase activity in the same samples. *n* = 3 experiments. **e**–**i** hNPCs were treated with DMSO or BBGD for 48 h. **e** GAPDH activity in the cell lysates. The same number of cells were seeded prior to treatment and the GAPDH activity was further normalized by total protein mass. *n* = 3 experiments. **f** Western blots probed for GAPDH and reprobed for ACTb as a loading control. *n* = 3 experiments. **g** qRT-PCR analysis of mRNA enrichment by RNA immunoprecipitation (RIP) with control IgG or an anti-GAPDH antibody (normalized to the total RNA input). *n* = 3 experiments. **h** qRT-PCR analysis of the relative distribution of the *HIF1a* and *c-MYC* mRNAs across all fractions from polysome profiling. **i** Quantification of the relative enrichment of *HIF1a* and *c-MYC* mRNAs in the heavy polysome fractions. Each point corresponds to the value of each fraction normalized to the total *HIF1a* or *c-MYC* mRNA. *n* = 3 experiments. Data are presented as mean values ± SEM and analyzed using two-tailed, unpaired students *t*-test. **p < 0.01, ns = *p* > 0.05. Source data and *p*-values are provided as a “Source Data file”.

To test this idea, we first asked whether GAPDH interacts with *Notch1* 3’UTR. We performed an RNA-electrophoretic mobility shift assay (REMSA) on in vitro transcribed and radioactively-labelled *Notch1* 3ʹUTR with different amounts of recombinant GAPDH (rGAPDH) protein, followed by the separation of RNA-protein complexes on native polyacrylamide gels. Multiple shifted bands of labelled RNA were detected in the presence of rGAPDH in a dose-dependent manner, indicating a direct interaction between GAPDH and the *Notch1* 3ʹUTR (Fig. 4a). This interaction was specific, as an excess of unlabeled *Notch1* 3ʹUTR RNA out-competed the binding of rGAPDH and abolished shifted bands of labelled *Notch1* 3ʹUTR RNA, while yeast tRNA was unable to out-compete the binding of rGAPDH to *Notch1* 3ʹUTR RNA (Fig. 4b).

We next asked whether the AU-rich region within the *Notch1* 3ʹUTR mediates this interaction. We repeated the REMSA using an in vitro synthesized *Notch1* 3ʹUTR containing a deletion of the AU-rich region and found no shifted bands in the presence of rGAPDH (Fig. 4c). Moreover, this mutant form of *Notch1* 3ʹUTR was unable to compete for the binding of rGAPDH to the full-length *Notch1* 3ʹUTR (Fig. 4c). Given that the AU-rich region in the *Notch1* 3ʹUTR can be bound by rGAPDH and was critical for the translational suppression of *Notch1* mRNA in cortical precursors (Fig. 3j, l), we speculated that GAPDH could suppress *Notch1* mRNA translation. To test this, we co-transfected the *Notch1* 3ʹUTR luciferase reporters into the human embryonic kidney (HEK) 293 cells with plasmids overexpressing GAPDH or EGFP as a control. Indeed, the luciferase assay showed that ectopic expression of *GAPDH* caused a 30% decrease in the luciferase activity from full-length *Notch1* 3ʹUTR, but not the mutant 3’UTR lacking the AU-rich region (Fig. 4d), suggesting that GAPDH acts as a translational repressor on the *Notch1* 3’UTR via the AU-rich region.

### Methylglyoxal engages GAPDH to suppress *Notch1* mRNA

Our data suggest that the translation of *Notch1* mRNA is controlled by its interaction with GAPDH. It is known that methylglyoxal can post-translationally modify GAPDH, and this modification inhibits its enzymatic activity for glycolysis^17^. Therefore, we explored the model that methylglyoxal modulates the dual function of GAPDH in NPCs by altering its enzymatic activity and subsequently engaging it as an RBP to bind *Notch1* mRNA, leading to translational repression. To test this, we first assessed GAPDH activity in hNPCs. Following BBGD treatment, we found that while GAPDH protein levels remained unchanged, GAPDH enzymatic activity was suppressed by approximately 80% (Fig. 4e, f).

We next asked if the suppression of GAPDH enzymatic activity induced by BBGD could lead to enhanced interaction between GAPDH and *NOTCH1* mRNA. We performed RNA immunoprecipitation (RIP) using antibodies against GAPDH or control isotype IgG from hNPCs extracts treated with or without BBGD. qRT-PCR analysis of immunoprecipitated RNA showed that the GAPDH antibody was enriched for *NOTCH1* mRNA as well as other known GAPDH target mRNAs encoding regulators of NPC fate decision, including *HIF1a*^39,40^ and *c-MYC*^41^ (Fig. 4g). The interaction was specific, as these target mRNAs were not immunoprecipitated by control IgG, and the GAPDH antibody was not able to immunoprecipitate *NESTIN* mRNA or 18 S RNA used as negative controls (Fig. 4g). Interestingly, following BBGD treatment, the amount of GAPDH-enriched *NOTCH1* mRNAs, but not *HIF1a* or *c-MYC* mRNAs, were markedly increased, suggesting the existence of functional selectivity (Fig. 4g). In agreement with this observation, we found that *HIF1a* and *c-MYC* mRNAs were actively engaged in translation regardless of BBGD treatment (Fig. 4h, i).

### GAPDH knockdown rescues methylglyoxal-induced NPC depletion

**Fig. 5:**
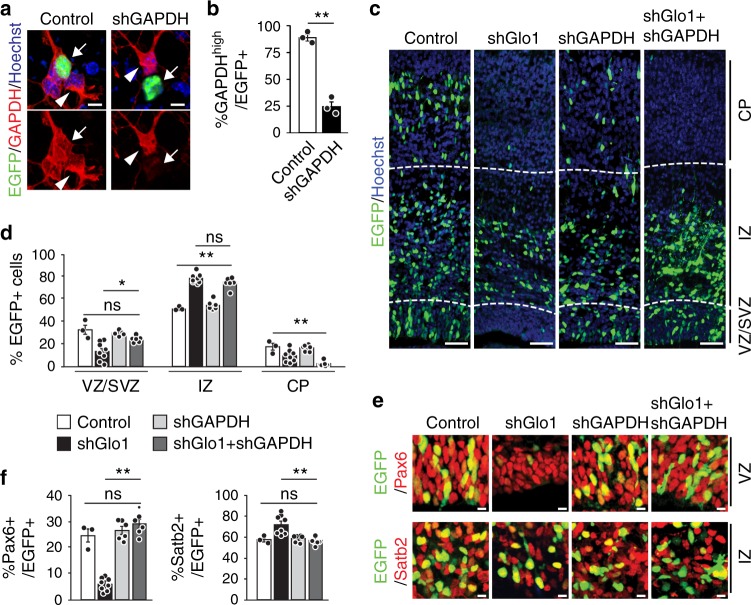
Reducing GAPDH abundance partially rescues the NPC depletion induced by excessive methylglyoxal. **a**, **b** Cultured E12.5 cortical precursors were co-transfected with EGFP and control or GAPDH shRNAs (shGAPDH) for three days, immunostained for EGFP (green) and GAPDH (red) (**a**) and quantified for EGFP-positive cells with detectable GAPDH (**b**). Arrows and arrowheads denote EGFP-positive and -negative cells, respectively. unpaired *t*-test; *n* = 3 experiments. **c**–**f** E13.5 cortices were co-electroporated with control or *Glo1* shRNAs together with or without a shRNA against GAPDH, and coronal cortical sections were analyzed three days later. **c** Images of electroporated sections immunostained for EGFP (green). Dotted white lines denote boundaries between VZ/SVZ, IZ and CP. **d** Quantification of sections as in **c** for the relative location of EGFP+ cells. *n* = 3 (Control), 8 (shGlo1), 6 (shGAPDH) and 6 (shGAPDH + shGlo1) embryos. **e** Images of the VZ or IZ of electroporated sections that were immunostained for EGFP (green) and Pax6 (red, top panels) or Satb2 (red, bottom panels). **f** Quantification of sections as in **e** for the proportion of EGFP+ cells that were also positive for Pax6 or Satb2. *n* = 3 (Control), 8 (shGlo1), 6 (shGAPDH) and 6 (shGAPDH + shGlo1) embryos. Sections in **a** and **c** were counterstained with Hoechst 33258 (blue). Scale bars, 100 µm in **c**, 10 µm in **a** and **e**. Data are presented as mean values ± SEM and analyzed using two-tailed, unpaired students *t*-test with Bonferroni correction. **p < 0.01, *p < 0.05, ns = *p* > 0.05. Source data and *p*-values are provided as a “Source Data file”.

The above results support a model whereby the modification of GAPDH by methylglyoxal engages it as an RBP to selectively suppress *Notch1* mRNA translation, leading to NPC depletion and premature neurogenesis. If the model is correct, a reduction in GAPDH abundance should ameliorate the impact of excessive methylglyoxal on NPCs in the developing cortex by releasing the repression on *Notch1* translation. To test this, we used a *GAPDH* shRNA that efficiently knocked down *GAPDH* expression in transfected cultured NPCs (Fig. 5a, b). We co-electroporated this shRNA with EGFP plus control or *Glo1* shRNAs into E13.5 cortices. Analysis 3 days later showed that while *Glo1* knockdown caused a robust reduction in EGFP+ cells in the VZ/SVZ and CP, concurrent knockdown of *GAPDH* and *Glo1* partially rescued the distribution of EGFP+ cells in the VZ/SVZ but not the CP (Fig. 5c, d). Moreover, *GAPDH* knockdown normalized the proportions of EGFP+ cells that were also positive for Pax6 or Satb2 (Fig. 5e, f). Together, these data suggest that methylglyoxal controls NPC homeostasis, at least in part, by engaging GAPDH as an RBP to regulate Notch signalling.

## Discussion

The coordination of gene expression and metabolism is essential for NPC homeostasis^2^. While much progress has been made to understand the gene networks that direct metabolic transitions during neurogenesis^42,43^, little is known about how metabolic cues signal back to gene expression programs to co-ordinate NPC self-renewal and differentiation. Our results reveal a mechanism mediating the metabolic control of gene expression in NPC homeostasis. We show that the intermediate metabolite methylglyoxal regulates the expression of the Notch1 receptor through the RBP function of the glycolytic enzyme GAPDH. As such, the metabolic signal is coupled to the translational control of Notch signalling to regulate the balance of NPC self-renewal and differentiation (Supplementary Fig. 4m).

Accumulating evidence suggests that intermediate metabolites are critical players in the crosstalk between metabolism and gene expression for stem-cell fate decision^2,42,43^. For example, two well-studied intermediates in the tricarboxylic acid (TCA) cycle, α-ketoglutarate, and Flavin adenine dinucleotide, function as cofactors of histone and DNA demethylases to epigenetically modulate global transcription for stem-cell maintenance^4,15,44^. In NPCs, however, glycolysis is the major source of energy, producing a plethora of intermediate metabolites, whose roles as signalling molecules in gene regulation and NPC homeostasis are poorly understood. Our results show that methylglyoxal, a glycolytic intermediate derived from G3P^21^, can modulate the translation of *Notch1* mRNA and the activity of Notch signalling, to co-ordinate NPC self-renewal and differentiation. Like other small signalling molecules, the magnitude of methylglyoxal signalling is likely to be determined by its steady-state concentration as the result of synthesis and degradation. Although high glycolytic rates in NPCs inevitably increase the production of methylglyoxal, high expression levels of *Glo1* in NPCs keep its concentration low (Fig. 1 and Supplementary Fig. 1), which permits active Notch signalling in NPCs. Therefore, it is likely that the rate of methylglyoxal clearance by Glo1 rather than synthesis from glycolysis dictates its signalling activity as a metabolic feedback control of NPC fate decision. This idea is supported by our findings that manipulation of Glo1 activity in NPCs had a strong impact on Notch signalling and NPC homeostasis (Figs. 1–3, Supplementary Fig. 1–4) while increasing glycolytic influx did not affect intracellular methylglyoxal levels (Supplementary Fig. 4d) and *NOTCH1* expression. However, how Glo1 activity is instructed by glycolysis to modulate intracellular methylglyoxal levels remains elusive.

It should be noted that the crosstalk mediated by methylglyoxal likely operates at multiple levels of gene regulation. In this regard, methylglyoxal has been recently shown to change histone modification and alter the epigenetic control of gene transcription^37^. Furthermore, methylglyoxal can also act directly on transcription factors to allow transcriptional regulation of specific genes in mammalian endothelial cells^21,38^. In NPCs, we found that methylglyoxal regulated *Notch1* receptor mRNA at the translational level (Fig. 3), suggesting a role of methylglyoxal in gene regulation beyond transcriptional regulation. A recent study in human umbilical vein endothelial cells corroborates this notion, showing that methylglyoxal affects the regulation of cell adhesion molecule-1 at the post-transcriptional level^45^. Given the diverse mechanisms in different contexts, it is possible that multiple stemness-related pathways may also contribute to the effect of methylglyoxal on NPC fate decision. Nonetheless, two lines of evidence suggest the presence of selectivity and indicate that methylglyoxal acts, at least in part, through Notch signalling. First, methylglyoxal induces translational suppression of Notch1 receptor but not HIF1a and c-MYC, both of which are critical regulators of NPC biology^39–41^ (Figs. 3, 4). Second, reactivation of Notch signalling results in a phenotypic rescue of *Glo1* knockdown (Fig. 2).

How do NPCs sense metabolic signals to co-ordinate translational regulation? One well-studied regulator involved in metabolic sensing and mRNA translation is the mechanistic (mammalian) target of rapamycin (mTOR). By interacting with sensor proteins, mTOR senses nutrients and amino acids, in part by coordinating global protein synthesis through cap-dependent translational machinery^46–48^. Nonetheless, how intermediate metabolites are sensed to regulate the translation of specific mRNA targets is not clear. Our results on methylglyoxal suggest a plausible mechanism for intermediate metabolite sensing and signalling, involving post-translational conjugation on RNA-binding metabolic enzymes. First, methylglyoxal is known to react with side chains, such as lysine and arginine to change protein function^21,22,36,49^. Although this reaction is thought to be enzymatically independent, the conjugation is target-specific in live cells through mechanisms that are still unclear. In the case of histones, methylglyoxal is conjugated selectively to the histone H3 and H4 families but not to H2, leading to changes in epigenetic modification and transcription^37^. The modification also occurs on some glycolytic enzymes, such as GAPDH, altering their enzymatic activity (Fig. 4)^17,50^. Interestingly, another glycolytic intermediate 1,3-BPG has also been found to modify metabolic enzymes and modulate their activity, suggesting that conjugation may be a common mechanism by which intermediates can be sensed to initiate signalling^51^.

Second, numerous metabolic enzymes can act as target-specific RBPs^52^. This dual function was initially observed in only a few enzymes, but recent mRNA-bound proteomic studies have identified a much broader class of metabolic enzymes involved in various pathways such as in glycolysis and the TCA cycle^11,12^. Our understanding of the functional roles of these dual role enzymes in RNA regulation under different biological contexts is still minimal. In NPCs, we found that GAPDH can bind to the 3ʹUTR of *Notch1* mRNA and suppress its translation (Figs. 3, 4). This binding was mediated by an AU-rich region, consistent with previous reports that GAPDH recognizes AU-rich sequences^10,18^. Importantly, we found that GAPDH contributed to NPC fate decision, at least in part by coordinating Notch signalling through its RNA-regulatory activity (Fig. 5). This finding represents a previously unrecognized route of regulation for NPC fate decision by metabolic enzymes. It should be noted that in addition to RNA- and glycolysis-related activities, GAPDH is known to perform other roles^13^. For example, it is known that upon cellular stress, GAPDH is translocated into the nucleus and triggers a signaling cascade resulting in apoptosis^53^. Therefore, it is possible that the changes in NPC homeostasis induced by *GAPDH* knockdown may reflect a combinatorial effect on multiple mRNA targets and different biological processes. Further studies are required to dissect the contribution of each target and process.

Third, the RNA-regulatory activity of metabolic enzymes is related to their enzymatic functions. For example, when GAPDH is dismissed from the glycolytic metabolism in T cells, its RBP function as a translational repressor of interferon γ is engaged, and when GAPDH participates in glycolysis, it no longer suppresses interferon γ translation^10^. Consistently, we found that inhibition of GAPDH enzymatic activity by methylglyoxal significantly enhanced its binding to *Notch1* mRNA for translational repression (Fig. 4). A decrease in GAPDH abundance, and thus less GAPDH to suppress *Notch1* mRNA, reversed the impact of methylglyoxal on NPC homeostasis (Fig. 5). These data support the idea that GAPDH is engaged as an RBP when its enzymatic activity is inhibited. Although our study reveals a functional switch of GAPDH induced by methylglyoxal in NPCs, how GAPDH enzymatic activity modulates its RNA-binding ability still requires further studies. Taken together, our findings in this study demonstrate a regulatory node that utilizes glycolytic metabolites to co-ordinate gene expression for NPC homeostasis.

## Methods

### Animals

All animal use was approved by the Animal Care Committees of the Hospital for Sick Children and the University of Calgary in accordance with the Canadian Council of Animal Care policies. Female CD1 mice (8–12 weeks old), purchased from Charles River Laboratory, were used for all animal experiments. Mice were housed in groups of 1–5/cage, at the temperature of 24 °C under a 12 h light-dark cycle with free access to food and water. For Glo1 inhibition in vivo, E13.5 embryos were used for the injection of 100 μM S-p-Bromobenzylglutathione cyclopentyl diester (BBGD) (Sigma) to lateral ventricles together with in utero electroporation.

### Cell culture

H9 hESCs were obtained from the National Stem Cell Bank (WiCell, Madison, WI) and cultured with mTeSR1 culture medium (StemCell Technologies) on BD hESC-qualified matrigel. Pluripotent stem-cell work was approved by the Canadian Institutes of Health Research Stem Cell Oversight Committee and The Hospital for Sick Children Research Ethics Board. An embryoid body (EB)-based method was used for the induction of NPCs^54^. EBs were made by treatment of hESCs with 2 mg ml^^−1^ collagenase type IV, and cultured in DMEM/F12 media supplemented with 20% KSR, 1x non-essential amino acid (NEAA), 1x beta-mercaptoethanol, 1x penicillin-streptomycin, 2.5 μM dorsomorphin, and 5 μM SB431542. After 4 days, the EBs were plated on Matrigel-coated dishes and cultured in induction media (DMEM/F12 media, N2 supplement, NEAA and 20 ng ml^^−1^ bFGF) for eight days. Cells were then fed with induction media plus B27 supplement every other day. After 1-week, neural rosettes were isolated by Dispase (StemCell Technologies) treatment and re-plated on Matrigel-coated plates and maintained in induction media for up to four passages. Briefly, HEK 293 cells were maintained in DMEM (Gibco) supplemented with 10% fetal bovine serum and 1% penicillin/streptomycin^55^.

### Plasmids and reagents

The pEF1α-EGFP plasmid expressing nuclear EGFP has been previously reported^56^. Glo1 and control shRNAs in the pSUPER vector were published previously^20^. The shRNAs against human GLO1 (GATGGCTACTGGATTGAAA) were cloned into the pSUPER vector. The shRNA against mouse GAPDH was obtained from EZbiolab. To generate luciferase reporter plasmids, the 3’UTR of *Notch1* mRNA was amplified from genomic DNA by PCR and cloned into the pmirGLO vector (Promega) downstream of the Firefly Luciferase gene. The AU-rich element (141–367 of 3’UTR) was deleted to generate the *Notch1* 3’UTR-dARE plasmid. A Renilla Luciferase, serving as the internal control, is expressed independently from the same pmirGLO plasmid. The pCAG-mGFP (#14757), CBFRE-EGFP (#17705) and 3XFlagNICD1 (#20183) plasmids were obtained from Addgene. Myc-DDK-GAPDH was obtained from the Origene. Human GLO1 cloned into lentiviral vector pLX304, from ORFeome initiative, was acquired from DNASU. All clones were verified by sequencing. Recombinant rabbit GAPDH was purchased from Sigma.

### Antibodies

The primary antibodies used were mouse anti-GFP (Invitrogen—1:1000), rabbit anti-GFP (Abcam—1:5000), chicken anti-GFP (Millipore—1:1000), mouse anti-SatB2 (Abcam—1:400), mouse anti-βIII-tubulin (Covance—1:1000), rabbit anti-βIII-tubulin (Covance—1:1000), rabbit anti-Pax6 (Covance—1:2000), mouse anti-Pax6 (Abcam—1:250), rabbit anti-GAPDH (Sigma—1:1000), rabbit anti-Tbr2 (Abcam—1:500), rabbit anti-Erk (Santa Cruz Biotechnology—1:1000), rabbit anti-Notch1 (Abcam—1:250), rabbit anti-RFP (MBL—1:1000), mouse anti-Actin (Sigma—1:2000), mouse anti-MG-H1 (Cell Biolabs—1:50), mouse anti-GAPDH (Abcam—1:1000), rabbit anti-GLO1 (Abcam 1:500), mouse anti-puromycin (Kerafast—1:1000). The donkey anti-mouse and anti-rabbit Alexa 488, 555 and 647-conjugated secondary antibodies were obtained from ThermoFisher and used at 1:500 dilutions. HRP-conjugated goat anti-mouse, anti-rabbit or anti-chicken secondary antibodies were purchased from ThermoFisher and used at 1:5000 dilutions. NIR-conjugated goat anti-mouse and anti-rabbit secondary antibodies were acquired from LI-COR and used at 1:25,000 dilutions.

### Lentiviral particles, transduction, and transfections

Lentiviruses were produced in HEK293T cells using the third generation packaging system (pMDG.2, pRSV-Rev, and pMDLg/pRRE). Supernatant containing viral particles were collected 48 h post-transfection, filtered and concentrated by 91,000 × *g* centrifugation for 2 h at 4 °C. For transduction, hNPCs were incubated overnight supplemented with the concentrated virus. hNPCs were transfected Lipofectamine 2000 (Invitrogen) according to manufacturer’s instructions. HEK293 cells were transfected with polyethylenimine.

### RNA immunoprecipitation

hNPCs were seeded in two 10 cm dishes and grown overnight. Cells were then washed in ice-cold PBS. RNA immunoprecipitations were carried out using EZ-Magna RIP-RNA Immunoprecipitation Kit (Millipore) according to the manufacturer’s instructions. Five micrograms of antibody rabbit anti-GAPDH (Sigma), was used per reaction. Five micrograms of rabbit isotype IgG antibody was used as negative control. Target transcripts were detected by qRT-PCR and plotted as % of Input.

### Methylglyoxal assay

Methylglyoxal concentration was determined using the methylglyoxal colorimetric assay kit (BioVision) according to the manufacturer’s instructions. To examine methylglyoxal levels in tissues, cortices were dissected from E13.5 embryonic brain, lysed with PBS/0.2% Triton-X and subjected to DMSO or BBGD treatment (50 μM) for 3 h. For cultured hNPCs, 5 × 10^5^ cells were lysed in 100 μl of PBS/0.1% Triton-X and 20 μl or serial dilutions used to determine methylglyoxal concentration changes using the same method as above.

### Fluorescence-activated cell sorting

For assessment of *Notch1* mRNA in vivo, a nuclear EGFP plasmid and control or *Glo1* shRNAs were in utero electroporated into different E13.5 embryos from the same mother. After 2 days, EGFP+ cortices from embryos of each group were dissected, pooled, and dissociated into single-cell suspensions in ice-cold HBSS. Dissociated cells were filtered through a 40 µm cell strainer to obtain suspended single cells for FACS using a BD FACS Aria II cell sorter (the University of Calgary Flow Cytometry Facility). The EGFP signal was detected at a 530/30 nm bandpass using a 488 nm laser, and dead cells were stained with propidium iodide and excluded from the analysis. EGFP + cells were sorted into ice-cold FBS, followed by the centrifugation for 5 min at 200 × *g* at 4 °C and total RNA analysis.

### Quantitative real-time PCR and immunoblotting

Total RNA was isolated from sorted mouse embryonic cerebral cortical cells or hES-derived NPCs using Trizol (ThermoFisher) according to the manufacturer’s instruction. cDNA was synthesized from 500 ng of total RNA using SuperScript III reverse transcriptase kit (ThermoFisher) with random hexamer primers according to manufacturer’s instructions. qRT-PCR was performed using SYBR Select PCR Master Mix (ThermoFisher) with amplification for 40 cycles with annealing temperature at 60 °C, using ViiA7 Real-Time PCR system (ThermoFisher). All primers used in this study are described in Table 1. Fold changes were calculated by the 2^-(∆∆Ct)^ method. For immunoblotting, hNPCs were lysed with radioimmune precipitation assay (RIPA) buffer (25 mM Tris-HCl, pH 7.6, 150 mM NaCl, 1% Nonidet P-40, 1% sodium deoxycholate, and 0.1% SDS). Equivalent protein mass was loaded on SDS-PAGE and transferred to Nitrocellulose membrane Hybond ECL (GE HealthCare). HRP-conjugated secondary antibodies (Invitrogen) were used, and the membranes were developed with SuperSignal West Pico Chemiluminescent Substrate (Pierce). Images acquired using ChemiDoc MP (BioRad) and quantified using software Imagelab v5.2.1 (BioRad). Cultured HEK293 cells were lysed with 1x sample buffer with 1 mM dithiothreitol (DTT), boiled for 8 minutes, and analyzed with SDS-PAGE^20^.

**Table 1:** Primers used in this study.

Gene	Primer	Sequence
Mouse *Notch1*	Forward	GATGGCCTCAATGGGTACAAG
	Reverse	TCGTTGTTGTTGATGTCACAGT
Mouse *Nestin*	Forward	CCCTGAAGTCGAGGAGCTG
	Reverse	CTGCTGCACCTCTAAGCGA
Mouse *Hes1*	Forward	TCAACACGACACCGGACAAAC
	Reverse	ATGCCGGGAGCTATCTTTCTT
Mouse *Hey1*	Forward	CCGACGAGACCGAATCAATAAC
	Reverse	TCAGGTGATCCACAGTCATCTG
Mouse *18* *S*	Forward	GTAACCCGTTGAACCCCATT
	Reverse	CCATCCAATCGGTAGTAGCG
Human *HES1*	Forward	TCAACACGACACCGGATAAAC
	Reverse	GCCGCGAGCTATCTTTCTTCA
Human *HES2*	Forward	CCAACTGCTCGAAGCTAGAGA
	Reverse	AGCGCACGGTCATTTCCAG
Human *HES5*	Forward	CTACCTGAAGCACAGCAAAG
	Reverse	AGCTTCATCTGCGTGTCG
Human *HEY1*	Forward	GTTCGGCTCTAGGTTCCATGT
	Reverse	CGTCGGCGCTTCTCAATTATTC
Human *HEY2*	Forward	AAGGCGTCGGGATCGGATAA
	Reverse	AGAGCGTGTGCGTCAAAGTAG
Human *MASH1*	Forward	CTGGACTTTACCAACTGGTTCTGA
	Reverse	CCTGCTTCCAAAGTCCATTCC
Human *NESTIN*	Forward	CTGCTACCCTTGAGACACCTG
	Reverse	GGGCTCTGATCTCTGCATCTAC
Human *GAPDH*	Forward	CATGAGAAGTATGACAACAGCCT
	Reverse	AGTCCTTCCACGATACCAAAGT
Human *18* *S*	Forward	GATGGGCGGCGGAAAATAG
	Reverse	GCGTGGATTCTGCATAATGGT
Renilla Luciferase	Forward	AAAGCGAAGAGGGCGAGAA
	Reverse	TGCGGACAATCTGGACGAC
Firefly Luciferase	Forward	TACCGGATTGCCCAAGGGCGTA
	Reverse	GAACATGCCGAAGCCGTGGTGA
c-MYC	Forward	GGAAAACAATGAAAAGGCCC
	Reverse	GTTGCATTTGATCATGCATTTG
HIF1a	Forward	AACATAAAGTCTGCAACATGGAAG
	Reverse	TTTGATGGGTGAGGAATGGG

### In utero electroporation

Expression constructs for nuclear EGFP or luciferases were co-electroporated with shRNAs or overexpression constructs at a 1:3 ratio, or when two additional plasmids were co-electroporated, at a 1:2:2 ratio for a total of 4 µg DNA or as indicated^55^. For detecting Notch signalling, control or *Glo1* shRNAs were co-electroporated with CBFRE-GFP and a plasmid expressing DsRed2 at a 2:2:1 ratio. Prior to injection, plasmids were mixed with 0.5% trypan blue. Following injection into lateral ventricles, the square electroporator CUY21 EDIT (TR Tech, Japan) was used to deliver five 50 ms pulses of 40-50 V with 950 ms intervals per embryo. Brains were dissected and analyzed 2 or 3 days post electroporation.

### Luciferase assay

Luciferase assays were carried out using Dual-Luciferase reporter kit (Promega) according to the manufacturer’s instructions. To examine activity and mRNA levels of luciferases in vivo, cortices were lysed 2 days after electroporation with pmirGLO plasmids together with control or *Glo1* shRNAs. For HEK293 cells, a plasmid expressing mouse GAPDH or EGFP was co-transfected with the luciferase reporter plasmids using Lipofectamine 2000 (Invitrogen) and incubated for 16–24 h. Transfected cortices or cells were lysed and subject to either luciferase assay or total RNA extraction for qRT-PCR analysis. The activity and mRNA levels of Renilla Luciferase were used as an internal control for normalization. The results presented are the average of at least three independent experiments.

### Immunostaining and histological analysis

For immunostaining of cortical sections, embryonic brains were dissected in ice-cold HBSS, fixed in 4% paraformaldehyde at 4 °C overnight, cryopreserved with 30% sucrose and cryosectioned coronally at 16 μm. Sections were blocked at room temperature with 5% BSA (Jackson Immunoresearch) in PBS with Triton-X, and incubated with primary antibodies in blocking buffer overnight at 4 °C, followed by incubation with appropriate secondary antibodies in blocking buffer at room temperature for 1 h. Nuclei were stained with Hoechst 33258 (Sigma). For the detection of BrdU on cortical sections, sections were first immunostained for indicated antibodies, followed by HCl treatment (1 N and 2 N) for 40 min, overnight incubation with anti-BrdU antibody at 4 °C and 1-h incubation with the secondary antibody at room temperature^20^.

### RNA-electrophoretic mobility shift assay—REMSA

For probe synthesis, the two fragments corresponding to the *Notch1* 3ʹUTR and *Notch1* 3ʹUTR dARE were PCR amplified and a T7 promoter sequence (TAATACGACTCACTATAGGG) was added upstream of the forward primer for in vitro transcription. In vitro transcriptions were carried out using MAXIscript T7 Kit (Invitrogen) and 50 μCi of 32P-labeled UTP 3000 Ci mMol^−1^ 10µCi µl^−1^ (EasyTide - BLU507T - 250µCi) according to manufacturer’s instructions. Corresponding probes without ^32^P-labeled UTP were also synthesized and used for specific competition control. After DNAse digestion, the probes were PAGE purified. For binding reaction and electrophoresis, 100.000 cpm of each probe was heated to 65 ° C for 10 min to denature and slowly cooled down to room temperature. Probes were combined with the recombinant GAPDH protein as indicated in binding buffer EBKM (25 mM HEPES pH 7.6, 5 mM MgCl2, 1.5 mM KCl, 75 mM NaCl, 6% sucrose, 1x complete protease inhibitor cocktail - Roche) plus 100 μg ml^−1^ BSA for 15 min at room temperature. Cold probes or 2.5 μg of tRNA were used as indicated. Two microlitres of a 50 mg ml^−1^ Heparin sulphate stock solution was then added to the reaction for an additional 15 min at room temperature. Samples were run in 4% native PAGE for 2 h at 200 V at 4 °C in 0.5x TBE buffer. The gel was dried, exposed in Phosphorimager, and images acquired in Typhoon FLA 9500 and analyzed using ImageQuant TL 8.1 software.

### GAPDH activity assay

GAPDH activity was detected using the KDalert GAPDH assay kit (Ambion) according to the manufacturer’s instructions. The same number of cells were seeded, four wells per condition, onto 96 well plates and treated with either BBGD or DMSO for 48 h. The results were normalized by total protein mass and expressed as a percentage of GAPDH activity relative to DMSO. The results presented are of three independent repeated experiments.

### Polysome fractionation

Human H9 NPCs (3 × 100-mm culture dish) were treated with DMSO or BBGD for 48 h, followed by a 10 min incubation with 100 μg ml^−1^ cycloheximide (CHX) (Sigma) at 37 °C. Cells were washed with ice-cold PBS containing CHX and harvested with lysis buffer (20 mM Tris-HCl pH 7.5, 100 mM KCl, 5 mM MgCl_2_, 1% Triton X-100, 1 mM DTT, 0.5% Sodium deoxycholate, 100 mg ml^−1^ CHX, 1% RNaseOUT) supplemented with protease inhibitors (Roche). After centrifugation at 13,000 × *g* for 5 min, the lysates were layered onto 10–50% continuous sucrose gradient and centrifuged at 39,000 rpm in a Beckman SW-41Ti rotor for 90 min at 4 °C. Each gradient was collected in 12 fractions^57–59^. RNA was extracted from all fractions and analyzed by qRT-PCR. Ten nanograms of synthetic luciferase transcript (NEB) was added to each fraction for normalization. Total RNA was then extracted using Trizol (Invitrogen). cDNA synthesis and qRT-PCR were carried out. The Ct values of transcripts were then normalized to spike-in luciferase transcript, and plotted as percent detected in each fraction.

### Puromycin metabolic incorporation assay

hNPC cultures in one well of a six-well plate treated with DMSO or BBGD were pulsed with 1 µM puromycin (Sigma) for 1 h and then processed for western blots as described above using an anti-puromycin antibody (EQ0001, KeraFast - 1:1000). The puromycin incorporation, representing protein synthesis during the pulse phase, was determined by measuring total lane signal from 15–250 kDa. Signals were quantified using Empiria (LI-COR), normalized to total protein levels using Revert total protein stain (LI-COR), and presented as percent change relative to DMSO control.

### Microscopy and quantification

For the analysis of embryonic brains with in utero electroporation, 3–4 anatomically matched sections per brain from at least three embryos of two to three independent mothers for each group were imaged with a ×20 objective on an Olympus IX81 fluorescence microscope equipped with a Hamamatsu C9100-13 back-thinned EM-CCD camera and Okogawa CSU X1 spinning disk confocal scan head^20^. Images were processed using Volocity software (Perkin Elmer). Pax6, Tbr2, and Hoechst staining were used to define the ventricular zone (VZ), subventricular zone (SVZ), and cortical plate (CP).

### Statistics

All data were expressed as the mean plus or minus the standard error of the mean (SEM). With the exception of the microarray data, statistical analyses were performed with a two-tailed Student’s *t*-test or, where relevant, ANOVA with Tukey or Dunnett’s post-hoc tests, unless otherwise indicated.

### Reporting summary

Further information on research design is available in the Nature Research Reporting Summary linked to this article.

## Supplementary information

Supplementary Information

Reporting Summary

## Data Availability

The source data underlying Figs. 1c, e, g; 2b–d, f, j; 3b–f, h-n; 4a–i; and 5b, d, f; and Supplementary Figs. 1c, f–h; 2a, c–f, h–k; 3b, c, e, f; and 4a–l are provided in the Source Data file.

## Source Data

Source Data
