# Supplementary material for: Methylglyoxal couples metabolic and translational control of Notch signalling in mammalian neural stem cells
Source: Nat Commun. 2020 Apr 24;11:2018. doi: 10.1038/s41467-020-15941-2 (PMC7181744; doi:10.1038/s41467-020-15941-2)
Supplement: Supplementary file 1 — Supplementary Information [file 41467_2020_15941_MOESM1_ESM.pdf]

Methylglyoxal couples metabolic and translational control of Notch signalling in mammalian neural stem cells

Rodrigues et al.

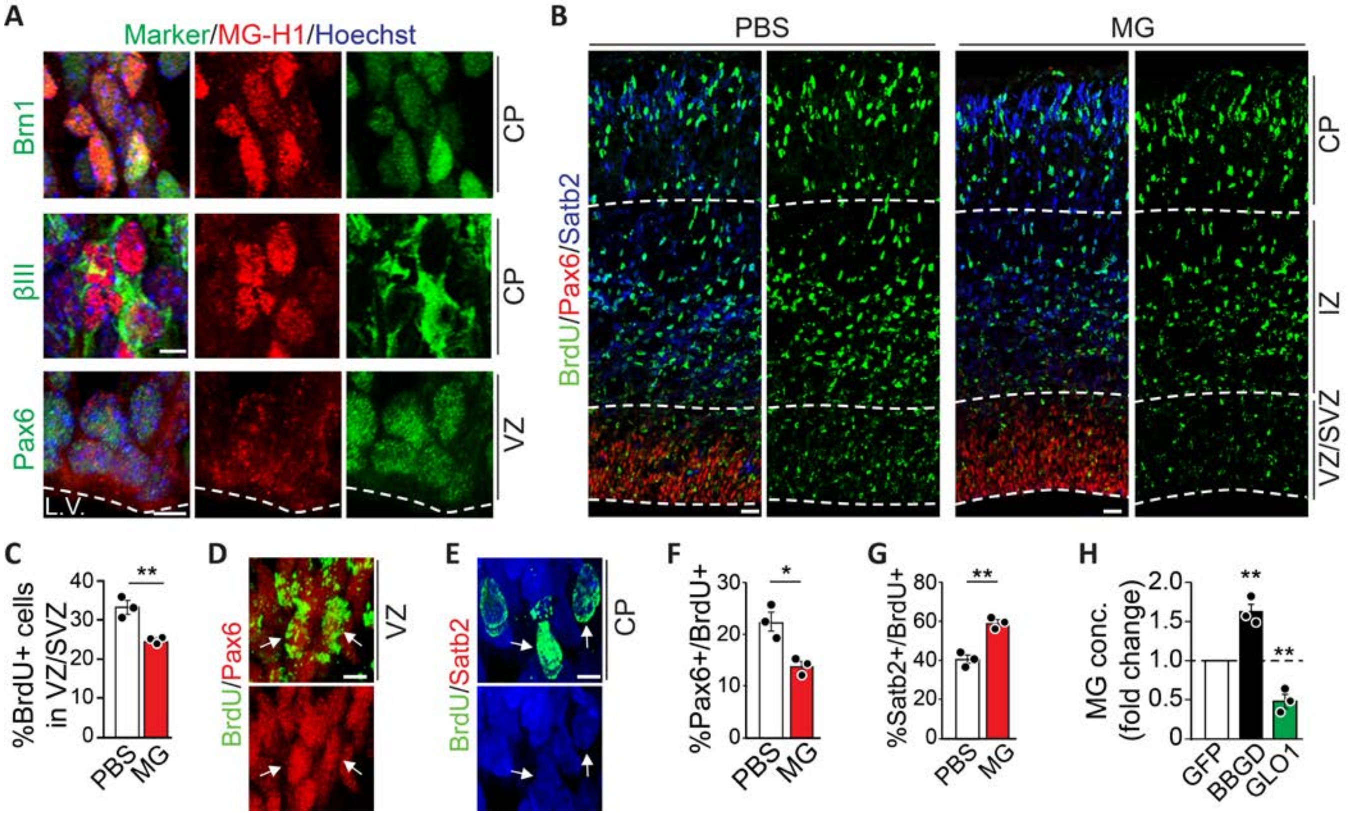

Supplementary Figure 1. Methylglyoxal regulates NPC homeostasis in the developing cortex. (**A**) Confocal images of the VZ and CP from E16.5 coronal cortical sections immunostained for MG-H1 (red) and Pax6 or Brn1 or βIII-tubulin (all green) and counterstained with Hoechst 33258 (blue). The ventricular surface of the cortex is labelled with dotted white lines. n = 2 experiments. (**B-G**) Pregnant mice at gestational day 13.5 were injected with BrdU 30 min before the injection of methylglyoxal or PBS into lateral ventricles of the embryos. Two days later, coronal cortical sections were immunostained for BrdU (green, **B**, **D**, **E**), Pax6 (red, **B**, **D**) and Satb2 (blue in **B**; red in **E**), and the proportion of BrdU+ cells in the VZ/SVZ was quantified (**C**). n = 3 embryos each. (**D, E**) High-magnification images of the VZ (**D**) or CP (**E**) from sections as in (**B**). Arrows denote double-labelled cells. (**F, G**) Quantification for the proportion of BrdU+ cells that were also positive for Pax6 (**F**) or Satb2 (**G**). n = 3 embryos each. (**H**) Relative methylglyoxal levels in hNPCs overexpressing human GLO1 or GFP as control or treated with BBGD. n = 3 experiments. LV: lateral ventricle; VZ: ventricular zone; SVZ: subventricular zone; IZ: intermediate zone; CP: cortical plate. Scale bars, 50 µm in (**B**), 10 mm in (**A**), (**D**) and (**E**). Data are presented as mean values +/- SEM and analyzed using two-tailed, unpaired students t-test with Bonferroni correction. ***p* < 0.01, **p* < 0.05. Source data and p-values are provided as a "Source Data file".

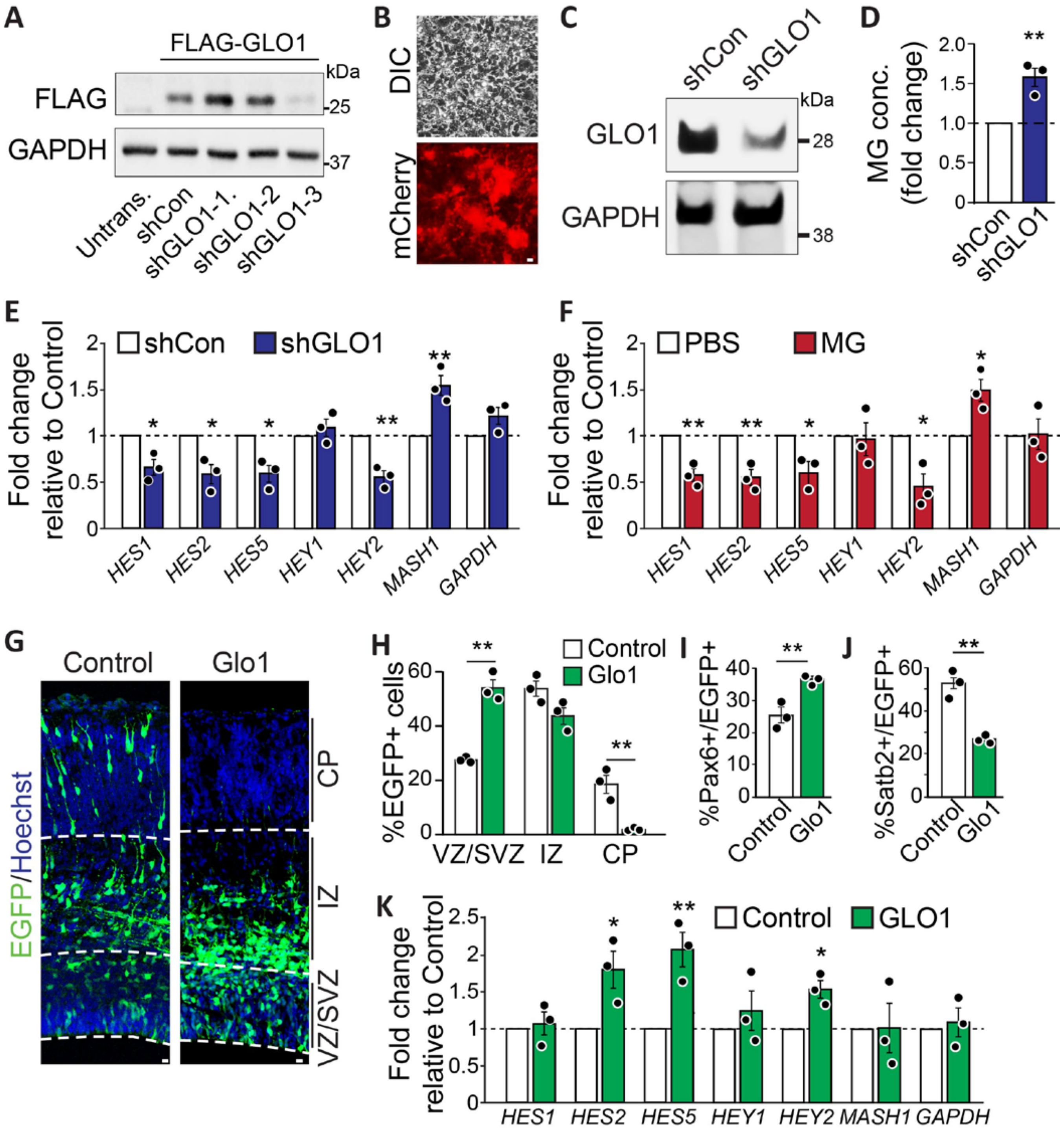

Supplementary Figure 2. Methylglyoxal alters Notch signalling to perturb NPC homeostasis. (**A**) Western blot analysis of HEK293 cells cotransfected with plasmids encoding FLAG-tagged human GLO1, with or without control (shCon) or one of three GLO1 shRNAs (shGLO1-1, shGLO1-2, and shGLO1-3), probed with anti-FLAG and reprobed with anti-GAPDH, from two experiments. Untransfected (Untrans.) cells served as a control. Molecular weight markers are indicated to the right.

(**B**) Representative images of hNPCs transfected with a plasmid expressing mCherry and cultured for 48 hr. Scale bar 10 µm (**C**) Western blot for GLO1 and GAPDH in hNPCs transfected with control (shCon) or GLO1 shRNAs (shGLO1-3), probed with anti-GLO1 and reprobed with anti-GAPDH, from 3 ??? experiments. Molecular weight markers are indicated to the right. (**D**) Relative methylglyoxal levels in hNPCs transfected with control or GLO1 shRNAs for 48 hr. n = 3 experiments. (**E**) qRT-PCR analysis of hNPCs transfected with control or GLO1 shRNAs for 48 hr. Values were normalized to the average of *ACTb* and 18S RNAs. n = 3 experiments. (**F**) qRT-PCR analysis of hNPCs treated with 500 µM methylglyoxal for 48 hr. Values were normalized to the average of *ACTb* and 18S RNAs. n = 3 experiments. (**G-J**) E13.5 cortices were electroporated with EGFP with or without Glo1, and coronal cortical sections were analyzed three days later. (**G**) Images of electroporated sections immunostained for EGFP (green). Dotted white lines denote the ventricular surface and boundaries between VZ/SVZ, IZ and CP. (**H**) Quantification of sections as in (**G**) for the relative location of EGFP+ cells. n = 3 embryos each. (**I, J**) Quantification for the proportion of EGFP+ cells that were also positive for Pax6 (**I**) or Satb2 (**J**). n = 3 embryos each. (**K**) qRT-PCR analysis of hNPCs transfected with a plasmid expressing human GLO for 48 hr. Values were normalized to the average of *ACTb* and 18S RNAs. n = 3 experiments. Scale bars, 10 µm. VZ: ventricular zone; SVZ: subventricular zone; IZ: intermediate zone; CP: cortical plate. Data are presented as mean values +/- SEM and analyzed using two-tailed, unpaired students t-test with Bonferroni correction. $^{**}p < 0.01$, $^{*}p < 0.05$. Source data and p-values are provided as a “Source Data file”.

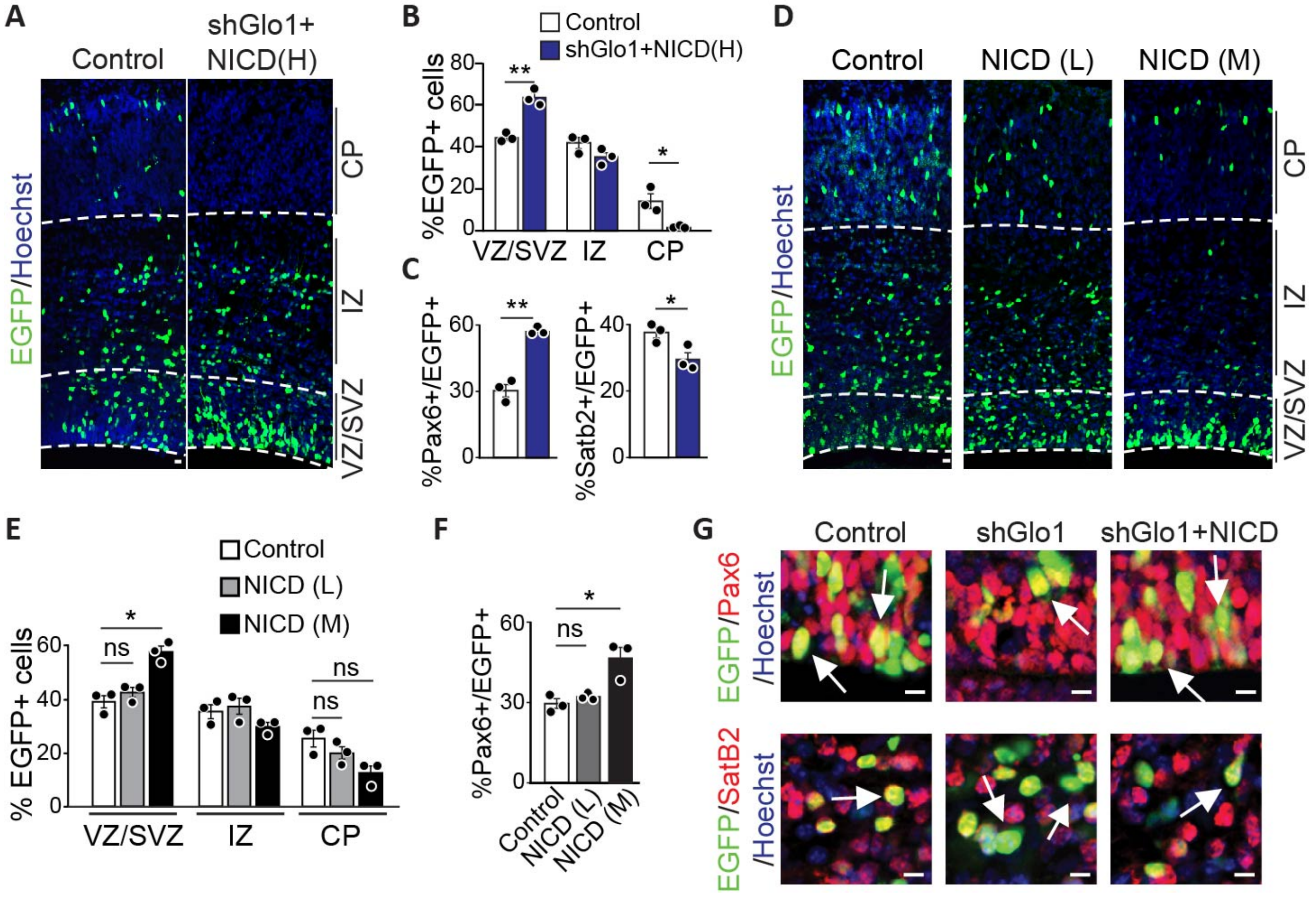

Supplementary Figure 3. Methylglyoxal-induced NPC depletion is rescued by reactivation of Notch signalling. (**A**-**C**) E13.5 cortices were analyzed three days following electroporation of EGFP or with Glo1 shRNA plus a high dose of plasmids (2 µg µl^-1) expressing NICD. (**A**) Representative images of sections immunostained for EGFP (green). (**B**) Quantification of sections as in (**A**) for the relative location of EGFP+ cells. n = 3 embryos each. (**C**) Quantification for the proportion of EGFP+ cells that were also positive for Pax6 or Satb2. n = 3 embryos each. (**D**-**F**) E13.5 cortices were electroporated with EGFP with or without a low (0.5 µg µl^-1, L) or medium (1 µg/µL, M) dose of plasmids expressing NICD and analyzed three days later. (**D**) Representative images of sections immunostained for EGFP (green). (**E**) Quantification of sections as in (**D**) for the relative location of EGFP+ cells. n = 3 embryos each. (**F**) Quantification for the proportion of EGFP+ cells that were also positive for Pax6. n = 3 embryos each. (**G**) Confocal images of E16.5 cortical sections co-electroporated with a low dose of plasmids (0.5µg µl^-1) expressing NICD and *Glo1* shRNA with EGFP at E13.5, immunostained for EGFP (green) and Pax6 (red, top panels) or Satb2 (red, bottom panels). Arrows denote double-labelled cells. Sections in (**A**), (**D**) and (**G**) were counterstained with Hoechst 33258 (blue). Scale bars, 10µm. Dotted white lines in (**A**) and (**D**) denote the ventricular surface and

boundaries between VZ/SVZ, IZ and CP. Scale bars, 10 µm. VZ: ventricular zone; SVZ: subventricular zone; IZ: intermediate zone; CP: cortical plate. Data are presented as mean values +/- SEM and analyzed using two-tailed, unpaired students t-test with Bonferroni correction. $^{**}p < 0.01$, $^{*}p < 0.05$, ns = $p > 0.05$. Source data and p-values are provided as a “Source Data file”.

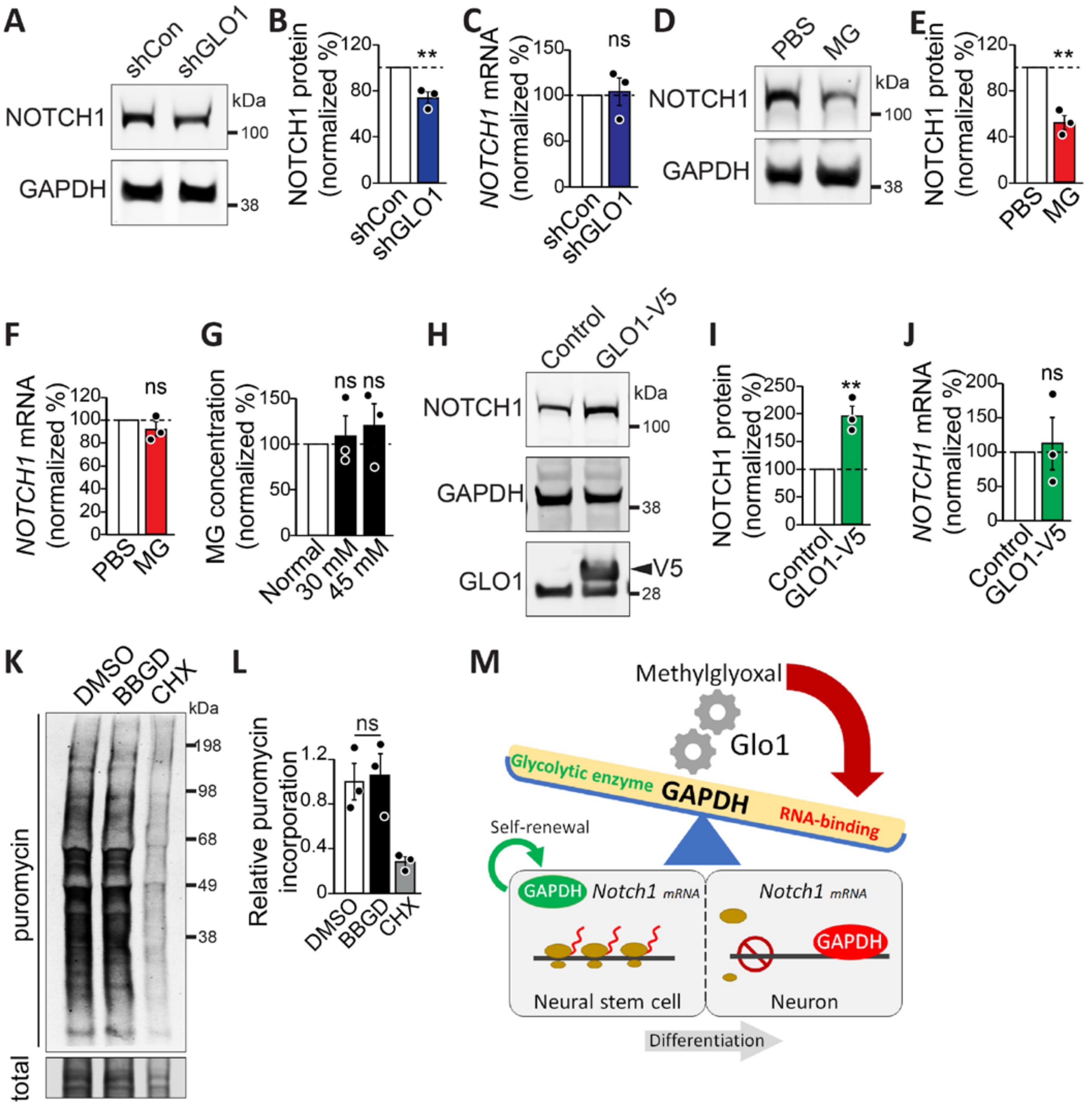

Supplementary Figure 4. Methylglyoxal alters NOTCH1 expression in cultured hNPCs. (**A-C**) Cultured hNPCs were transfected with control or GLO1 shRNAs for 48 hr. (**A**) Western blot analysis of hNPCs probed for NOTCH1 and GAPDH. Molecular weight markers are indicated to the right. (**B**) Quantification of NOTCH1 protein abundance from three independent experiments. Values were normalized to the levels of GAPDH. n = 3 experiments. (**C**) qRT-PCR analysis of hNPCs for *NOTCH1* mRNA. Values were normalized to the average of *ACTb* hNPCand 18S RNAs. n = 3 experiments. (**D-F**) hNPCs were treated with PBS or methylglyoxal for 48 hr. Western blot analysis of hNPCs probed for

NOTCH1 and GAPDH (**D**), and the quantification of NOTCH1 proteins (**E**). Values were normalized to the levels of GAPDH. n = 3 experiments. (**F**) qRT-PCR analysis of hNPCs for *NOTCH1* mRNA. Values were normalized to the average of *ACTb* hNPC and 18S RNAs. n = 3 experiments. (**G**) Relative methylglyoxal levels in hNPCs cultured under normal glucose (17.5 mM) or high glucose conditions (30 mM and 45 mM) for 48 hr. n = 3 experiments. (**H**-**J**) hNPCs were transduced to overexpress human GLO1 (GLO1-V5) for 48 hr. (**H**) Western blot analysis of transduced hNPCs probed for NOTCH1, GAPDH and GLO1. Molecular weight markers are indicated to the right. (**I**) Quantification of NOTCH1 protein abundance in transduced hNPCs. Values were normalized to the levels of GAPDH. n = 3 experiments. (**J**) qRT-PCR analysis of hNPCs for *NOTCH1* mRNA. Values were normalized to the average of *ACTb* hNPC and 18S RNAs. n = 3 experiments. (**K**, **L**) Metabolic incorporation assays using puromycin, from 3 experiments. The protein synthesis inhibitor cycloheximide (CHX) was used as negative control. Differences in puromycin incorporation levels were normalized by total protein mass. (**M**) A schematic model showing that an intermediate metabolite methylglyoxal induces the switch of GAPDH function from serving as a glycolytic enzyme to an RNA-binding protein that binds and suppresses the translation of *Notch1* mRNA. As such, the metabolic signal modulated by Glo1 is coupled to the translational control of pro-proliferative Notch signalling to regulate the balance of NPC self-renewal and differentiation. Data are presented as mean values +/- SEM and analyzed using two-tailed, unpaired students t-test with Bonferroni correction. $^{**}p < 0.01$, ns = $p > 0.05$. Source data and p-values are provided as a “Source Data file”.
