# Supplementary material for: Methylglyoxal couples metabolic and translational control of Notch signalling in mammalian neural stem cells
Source: Nat Commun. 2020 Apr 24;11:2018. doi: 10.1038/s41467-020-15941-2 (PMC7181744; doi:10.1038/s41467-020-15941-2)
Supplement: Supplementary file 2 — Reporting Summary [file 41467_2020_15941_MOESM2_ESM.pdf]

nature research

Corresponding author(s): Guang Yang

Last updated by author(s): Feb 1, 2020

# Reporting Summary

Nature Research wishes to improve the reproducibility of the work that we publish. This form provides structure for consistency and transparency in reporting. For further information on Nature Research policies, see Authors & Referees and the Editorial Policy Checklist.

## Statistics

For all statistical analyses, confirm that the following items are present in the figure legend, table legend, main text, or Methods section.

| n/a | Confirmed | |
|---|---|---|
| ☐ | ☒ | The exact sample size ($n$) for each experimental group/condition, given as a discrete number and unit of measurement |
| ☐ | ☒ | A statement on whether measurements were taken from distinct samples or whether the same sample was measured repeatedly |
| ☐ | ☒ | The statistical test(s) used AND whether they are one- or two-sided<br>*Only common tests should be described solely by name; describe more complex techniques in the Methods section.* |
| ☒ | ☐ | A description of all covariates tested |
| ☐ | ☒ | A description of any assumptions or corrections, such as tests of normality and adjustment for multiple comparisons |
| ☐ | ☒ | A full description of the statistical parameters including central tendency (e.g. means) or other basic estimates (e.g. regression coefficient) AND variation (e.g. standard deviation) or associated estimates of uncertainty (e.g. confidence intervals) |
| ☐ | ☒ | For null hypothesis testing, the test statistic (e.g. $F$, $t$, $r$) with confidence intervals, effect sizes, degrees of freedom and $P$ value noted<br>*Give P values as exact values whenever suitable.* |
| ☒ | ☐ | For Bayesian analysis, information on the choice of priors and Markov chain Monte Carlo settings |
| ☒ | ☐ | For hierarchical and complex designs, identification of the appropriate level for tests and full reporting of outcomes |
| ☒ | ☐ | Estimates of effect sizes (e.g. Cohen's $d$, Pearson's $r$), indicating how they were calculated |

*Our web collection on statistics for biologists contains articles on many of the points above.*

## Software and code

Policy information about availability of computer code

| | |
|---|---|
| Data collection | Imaged were taken with a 20X objective on an Olympus IX81 fluorescence microscope equipped with a Hamamatsu C9100-13 back-thinned EM-CCD camera and Okogawa CSU X1 spinning disk confocal scan head with Volocity software (Perkin Elmer, 6.0) or a FV3000 confocal microscope from Olympus. Images were processed using ImageJ v1.51w.<br>Western blot images were acquired on a BioRad ChemiDoc MP system using ImageLab 5.2.1 software, or LI-COR Odysssey using Image Studio 5.2 software and analyzed using Empiria Studio 1.2<br>REMSA images were taken using GE Typhoon FLA9500 Phosphorimager and analyzed using ImageQuant TL 8.1 software |
| Data analysis | Confocal microscope images were processed using ImageJ v1.51w with the FIJI plugin. Western blot images were analyzed using either ImageLab 5.2.1 or Empiria Studio 1.2 softwares.<br>FACS data was analyzed using BD FACSDiva 8.0.1 |

For manuscripts utilizing custom algorithms or software that are central to the research but not yet described in published literature, software must be made available to editors/reviewers. We strongly encourage code deposition in a community repository (e.g. GitHub). See the Nature Research guidelines for submitting code & software for further information.

## Data

Policy information about availability of data

All manuscripts must include a data availability statement. This statement should provide the following information, where applicable:

- Accession codes, unique identifiers, or web links for publicly available datasets
- A list of figures that have associated raw data
- A description of any restrictions on data availability

The source data underlying Figures 1C, E, G; 2B-D, F, J; 3B-F, H-N; 4A-I; and 5B, D, F;  and Supplementary Figures 1C, F-H; 2A, C-F, H-K; 3B, C, E, F; and 4A-L are provided in the Source Data file.

# Field-specific reporting

Please select the one below that is the best fit for your research. If you are not sure, read the appropriate sections before making your selection.

☒ Life sciences ☐ Behavioural & social sciences ☐ Ecological, evolutionary & environmental sciences

For a reference copy of the document with all sections, see nature.com/documents/nr-reporting-summary-flat.pdf

# Life sciences study design

All studies must disclose on these points even when the disclosure is negative.

| | |
|---|---|
| Sample size | No calculations were performed to predetermine sample-size. Sample sizes was determined to be adequate based on the magnitude and consistency of measurable differences between groups. >= 3 animals or experiments were used as indicated in the figure legends. |
| Data exclusions | no data excluded. |
| Replication | The experiments were repeated for 3 times unless otherwise indicated in the raw data file. All attempts at replication were successful. |
| Randomization | No randomization was used in this manuscript. The data reported in our experiments is not affected by sample allocation. |
| Blinding | The investigators were not blinded to experimental groups during data collection and analysis. Data reported for these experiments is not subjective. |

# Reporting for specific materials, systems and methods

We require information from authors about some types of materials, experimental systems and methods used in many studies. Here, indicate whether each material, system or method listed is relevant to your study. If you are not sure if a list item applies to your research, read the appropriate section before selecting a response.

## Materials & experimental systems

| n/a | Involved in the study | |
|---|---|---|
| ☐ | ☒ | Antibodies |
| ☐ | ☒ | Eukaryotic cell lines |
| ☒ | ☐ | Palaeontology |
| ☐ | ☒ | Animals and other organisms |
| ☒ | ☐ | Human research participants |
| ☒ | ☐ | Clinical data |

## Methods

| n/a | Involved in the study | |
|---|---|---|
| ☒ | ☐ | ChIP-seq |
| ☐ | ☒ | Flow cytometry |
| ☒ | ☐ | MRI-based neuroimaging |

## Antibodies

Antibodies used

Mouse anti-GFP 1:1000 Cat #A-11120 - https://www.thermofisher.com/antibody/product/GFP-Antibody-clone-3E6-Monoclonal/A-11120

Rabbit anti-GFP 1:5000 https://www.abcam.com/gfp-antibody-chip-grade-ab290.html

Chicken anti-GFP 1:1000 https://www.emdmillipore.com/CA/en/product/Anti-Green-Fluorescent-Protein-Antibody,MM_NF-AB16901

Mouse anti-SatB2 1:400 https://www.abcam.com/satb2-antibody-satba4b10-c-terminal-ab51502.html

Mouse anti-βIII-tubulin 1:1000 https://www.biolegend.com/en-us/products/purified-anti-tubulin-beta-3-tubb3-antibody-11580

Rabbit anti-βIII-tubulin 1:1000 https://www.biolegend.com/en-us/products/purified-anti-tubulin-beta-3-tubb3-antibody-11579

Rabbit anti-Pax6 1:2000 https://www.biolegend.com/en-us/products/purified-anti-pax-6-antibody-11511

Mouse anti-Pax6 1:250 https://www.abcam.com/pax6-antibody-ad238-ab78545.html

Rabbit anti-GAPDH 1:1000 https://www.sigmaaldrich.com/catalog/product/sigma/sab2108668?lang=en&region=CA&gclid=Cj0KCQiAvc_xBRCYARIsAC5QT9lKQVQHxinIpA_xlQsuQjCLPCL7lOvOQA0YF28qd2ktBGgzTbdkpTMaAhusEALw_wcB

Rabbit anti-Tbr2 1:500 https://www.abcam.com/tbr2-eomes-antibody-chip-grade-ab23345.html

Rabbit anti-Erk 1:1000 https://www.scbt.com/p/erk-1-2-antibody-h-72

Rabbit anti-Notch1 1:250 https://www.abcam.com/notch1-antibody-ab65297.html

Rabbit Anti-Notch1 Abcam ab65297 1:500 - https://www.abcam.com/notch1-antibody-ab65297.pdf

Rabbit anti-RFP 1:1000 https://ruo.mbl.co.jp/bio/e/dtl/A/?pcd=PM005

Mouse anti-MG-H1 1:50 https://www.cellbiolabs.com/anti-methylglyoxal-antibody

Mouse Anti-GAPDH antibody [mAbcam 9484] Abcam ab9482 1:1000 - https://www.abcam.com/gapdh-antibody-mabcam-9484-loading-control-hrp-ab9482.pdf

Rabbit Anti-GLO1 antibody [EPR8878] Abcam ab137098 1:500 - https://www.abcam.com/glo1-antibody-epr8878-ab137098.pdf

Mouse Anti-Puromycin (3RH11) Kerafast EQ0001 1:1000 - https://www.kerafast.com/productgroup/190/anti-puromycin-3rh11-antibody

Mouse anti-Actin Sigma A5316 1:2000- https://www.sigmaaldrich.com/catalog/product/sigma/a5316?lang=en&region=CA

Donkey anti-rabbit Alexa 488 ThermoFisher A21206 1:500- https://www.thermofisher.com/order/genome-database/generatePdf?productName=Rabbit%20IgG%20(H+L)%20Highly%20Cross-Adsorbed&assayType=PRANT&detailed=true&productId=A-21206

Donkey anti-mouse Alexa 488 ThermoFisher A21202 1:500 - https://www.thermofisher.com/order/genome-database/generatePdf?productName=Mouse%20IgG%20(H+L)%20Highly%20Cross-Adsorbed&assayType=PRANT&detailed=true&productId=A-21202

Donkey anti-rabbit Alexa 555 ThermoFisher A31572 1:500 - https://www.thermofisher.com/order/genome-database/generatePdf?productName=Rabbit%20IgG%20(H+L)%20Highly%20Cross-Adsorbed&assayType=PRANT&detailed=true&productId=A-31572

Donkey anti-mouse Alexa 555 ThermoFisher A31570 1:500 - https://www.thermofisher.com/order/genome-database/generatePdf?productName=Mouse%20IgG%20(H+L)%20Highly%20Cross-Adsorbed&assayType=PRANT&detailed=true&productId=A-31570

Donkey anti-mouse Alexa 647 ThermoFisher A31571 1:500 - https://www.thermofisher.com/order/genome-database/generatePdf?productName=Mouse%20IgG%20(H+L)%20Highly%20Cross-Adsorbed&assayType=PRANT&detailed=true&productId=A-31571

Donkey anti-rabbit Alexa 647 ThermoFisher A31573 1:500 - https://www.thermofisher.com/order/genome-database/generatePdf?productName=Rabbit%20IgG%20(H+L)%20Highly%20Cross-Adsorbed&assayType=PRANT&detailed=true&productId=A-31573

Goat anti-rabbit HRP ThermoFisher 65-6120 1:5000- https://www.thermofisher.com/antibody/product/Goat-anti-Rabbit-IgG-H-L-Secondary-Antibody-Polyclonal/65-6120

Goat anti-mouse HRP ThermoFisher 62-6520 1:5000 - https://www.thermofisher.com/antibody/product/Goat-anti-Mouse-IgG-H-L-Secondary-Antibody-Polyclonal/62-6520

IRDye® 800CW Goat anti-Rabbit IgG (H + L) LI-COR 926-32211 1:20,000 - https://www.licor.com/bio/reagents/irdye-800cw-goat-anti-rabbit-igg-secondary-antibody

IRDye® 800CW Goat anti-Mouse IgG LI-COR 926-32210 1:20,000 - https://www.licor.com/bio/reagents/irdye-800cw-goat-anti-mouse-igg-secondary-antibody

Validation

Validation of each antibody was provided by the manufacturer with each batch as shown in the list above. GLO1 antibody was internally validated using the KD and over-expression samples.

# Eukaryotic cell lines

Policy information about cell lines

Cell line source(s)

ATCC was the original source of HEK293 Cells (CRL-1573). hNPCs were derived from H9 hESCs obtained from the National Stem Cell Bank (WiCell).

Authentication

No cell line authentication was performed.

Mycoplasma contamination

our lab routinely test for mycoplasma contamination and we did not have contamination issues during the period of this study

| | |
|---|---|
| Commonly misidentified lines (See ICLAC register) | There were no commonly misidentified cell lines used in these experiments |

## Animals and other organisms

Policy information about studies involving animals; ARRIVE guidelines recommended for reporting animal research

| | |
|---|---|
| Laboratory animals | CD1 mice (8-12 week old), purchased from Charles River Laboratory, were used for all animal experiments. Mice were housed in groups of 1-5/cage, at the temperature of 24 °C under a 12 h light-dark cycle with free access to food and water. |
| Wild animals | No wild animals used |
| Field-collected samples | No samples collected from the field |
| Ethics oversight | All animal use was approved by the Animal Care Committees of the Hospital for Sick Children and the University of Calgary in accordance with the Canadian Council of Animal Care policies |

Note that full information on the approval of the study protocol must also be provided in the manuscript.

## Flow Cytometry

### Plots

Confirm that:

☒ The axis labels state the marker and fluorochrome used (e.g. CD4-FITC).

☒ The axis scales are clearly visible. Include numbers along axes only for bottom left plot of group (a 'group' is an analysis of identical markers).

☒ All plots are contour plots with outliers or pseudocolor plots.

☒ A numerical value for number of cells or percentage (with statistics) is provided.

### Methodology

| | |
|---|---|
| Sample preparation | EGFP+ cortices from embryos of each experimental group were dissected, pooled and dissociated into single-cell suspensions in ice-cold HBSS. Dissociated cells were filtered through a 40µm cell strainer to obtain suspended single cells. FACS was performed using a BD FACS Aria II cell sorter. The EGFP signal was detected at a 530/30nm bandpass using a 488nm laser, and dead cells were stained with propidium iodide and excluded. EGFP+ cells were sorted into ice-cold FBS, followed by the centrifugation for 5 minutes at 200xg at 4°C and total RNA analysis. |
| Instrument | BD FACS Aria II |
| Software | BD FACSDiva 8.0.1 |
| Cell population abundance | The total number of EGFP+ cells in post-sort fractions ranged from 11,000-51,000 cells per sample, with a purity of >83%. |
| Gating strategy | Gating strategy can be found in Data Source File. |

☒ Tick this box to confirm that a figure exemplifying the gating strategy is provided in the Supplementary Information.
